# Effects of drying temperature, mercerizing, and coating on the properties of Colombian Coir fibers and their interfacial adhesion with polylactic acid

**DOI:** 10.1038/s41598-025-18240-2

**Published:** 2025-09-02

**Authors:** Yarley Buelvas Arrieta, Linda Díaz Reyes, César Ávila-Díaz, Juan Altamiranda Suárez, Oswaldo Rivero-Romero, Jimy Unfried-Silgado

**Affiliations:** 1 Department of Mechanical Engineering, University of Cordoba, Montería, Córdoba Colombia; 2 Department of Mechanical Engineering, University of Antioquia, Medellín, Antioquia Colombia

**Keywords:** Coir fiber, Drying, Polymeric coating, Mercerization, Polylactic acid, Interfacial adhesion, Materials science, Mechanical engineering

## Abstract

In the present study, the influence of drying temperature, mercerization, and epoxy coating on the physicochemical and mechanical properties of Colombian CFs and their interfacial adhesion with a PLA matrix were studied. CFs were extracted from the mesocarp of Cocos nucifera husks by retting. The fibers were oven-dried at two drying temperatures, 40 °C and 90 °C. These samples were then mercerized and coated with epoxy resin. Pullout tests were performed to assess the bonding between the fibers and the PLA matrix. The results revealed that drying at 90 °C significantly enhanced fiber stiffness, yielding a Young’s modulus of 1.99 ± 0.48 GPa. Scanning electron microscopy (SEM) and Fourier transform infrared spectroscopy (FTIR) revealed that the mercerization treatment partially removed lignin from the surface of the fibers, increasing their roughness and facilitating mechanical interlocking. The subsequent epoxy resin coating introduced functional groups that can enhance the wettability of PLA. Compared with the untreated fibers, the combination of the mercerized and polymeric coatings resulted in a 62% increase in the Young’s modulus (3.57 ± 0.95 GPa) and a 43% increase in the tensile strength (108.43 ± 35.72 MPa). Additionally, single-fiber pull-out tests revealed that mercerized and coated fibers (CM-PLA) had double the interfacial shear strength (0.916 MPa) of untreated fibers, demonstrating improved fiber‒matrix adhesion.

**Supplementary Information:**

The online version contains supplementary material available at 10.1038/s41598-025-18240-2.

## Introduction

Lignocellulosic fibers (LFs) have gained interest as potential reinforcements in polymer matrix composites because of their low cost, renewable nature, biodegradability, and environmental friendliness^1,2^. In addition, LFs have an acceptable strength-to-weight ratio, making them a sustainable alternative to synthetic fibers^3^. LFs used in composite materials are commonly referred to as technical fibers and are mostly composed of interconnected elementary fibers that form fiber bundles^2,4^. These elementary fibers consist of several cell walls, where cellulose microfibrils are connected by amorphous lignin and hemicelluloses^2^. Consequently, the hierarchical microstructure and chemical composition of these materials determine their physical and mechanical properties. These features influence their potential as reinforcement materials in polymer matrix composites^5^. For instance, the presence of hydroxyl groups on LFs makes them incompatible with certain hydrophobic thermoplastics, such as polycaprolactone (PCL)^6^. Therefore, it is recommended that the fibers undergo appropriate surface treatment to ensure compatibility with polymer matrices^7^. Growing environmental concerns have led to increased interest among researchers in the use of LFs derived from agro-industrial waste^8^. Indeed, the incorporation of these fibers into composite materials has emerged as a promising strategy for waste management^9^. For example, LFs extracted from residues such as pineapple leaves, banana pseudostem, and coconut mesocarps have been used to make composites, even with 3D printing manufacturing techniques^10–12^. This is significant, as it enables the adoption of this technology to advance circular economy models^13^. Using LFs to reinforce polymer matrices has several advantages, including their abundance, biodegradability, and low cost. These advantages can reduce the overall cost of manufacturing composites and provide an opportunity for the valorization of agricultural residues^14^ Among LFs, Coir fiber (CF), derived from the mesocarp of *Cocos nucifera* fruit, stands out because of its high availability. It is widely used in various industrial applications in tropical and subtropical regions^15,16^. These fibers are characterized by low thermal conductivity and low bulk density^17^. In addition, it has a greater elongation at break than other natural fibers do because of its higher lignin content and microfibrillar angle^16^. This makes it an ideal choice for reinforcing polymer materials for multiple industrial applications^18^. Recently, CF has been used to produce composite boards in the construction industry, automotive parts, and fillers for crash helmets^19,20^. Polylactic acid (PLA) is a widely used thermoplastic polymer because of its biocompatibility, compostability, and ease of processing. These properties make it a viable alternative to traditional polymers, particularly in 3D printing applications^21–23^. Despite these advantages, the rigidity of its main chain structure can lead to brittleness and low elongation at break^22,24^. Therefore, the use of LFs as reinforcements in the PLA matrix has attracted considerable interest as a potential solution to these problems. Some works have reported that incorporating up to 30 wt% CF into PLA can increase the elongation of the polymer at break by up to 44%^25^. Nevertheless, one of the greatest challenges is the inherently poor interfacial adhesion between PLA and CF. A weak interface can hinder stress transfer between two materials^26^.

CF has been shown to enhance mechanical properties such as tensile, flexural, and impact strengths in polymers such as vinyl ester, epoxy, and polyester resins^27–29^. However, research on the use of CF as a reinforcement for PLA matrices remains limited. Few studies have focused on evaluating the effects of physical and chemical treatments on physicochemical, morphological, and mechanical properties and their interfacial compatibility with PLA. Among the physical treatments, drying is applied to prevent deterioration and extend the shelf-life of LFs^30^. Authors such as Martinelli et al.^31^ have demonstrated that optimizing drying temperatures can enhance the mechanical performance of CFs, improving both their strength and ductility. This aspect is particularly relevant because processes such as retting require the submergence of coconut husks in water to separate the fibers through the biological degradation of pectin^15^. On the other hand, chemical treatments such as mercerization are used to enhance interfacial adhesion^32^. This treatment removes surface impurities, increases fiber roughness, and enhances mechanical anchoring within the polymer matrix^33–35^. Consequently, mercerized fibers possess significantly higher interfacial shear strength (IFSS) than untreated fibers do^8^. However, prolonged exposure to alkaline solutions can cause fiber degradation^19^.

Other treatments, such as silane agents and plasma, have also been studied^36,37^. However, the feasibility of implementing these treatments for scalable use may be limited by costs, complex equipment, and labor-intensive processes. Another less explored alternative for the surface treatment of LFs is polymer coatings^38–40^. These coatings protect against moisture and chemically modify the surface of fibers^41^. Among the available options, epoxy resins (ERs) stand out because of their exceptional adhesive properties, as well as their chemical and thermal resistance, making them ideal modifiers for high-performance fiber/polymer composites^42^. Their implementation could address critical issues in PLA composites reinforced with CF, such as poor interfacial adhesion and thermal instability during high-temperature processing (~ 200 °C)^43^. Authors such as Sujaritjun et al.^44^ reported that the ER significantly improved interfacial bonding in PLA/CF composites. Additionally, Nuthong et al.^45^ reported that the use of flexible epoxy resins promotes the formation of a more ductile interface, which increases the material’s impact resistance.

**Fig. 1:**
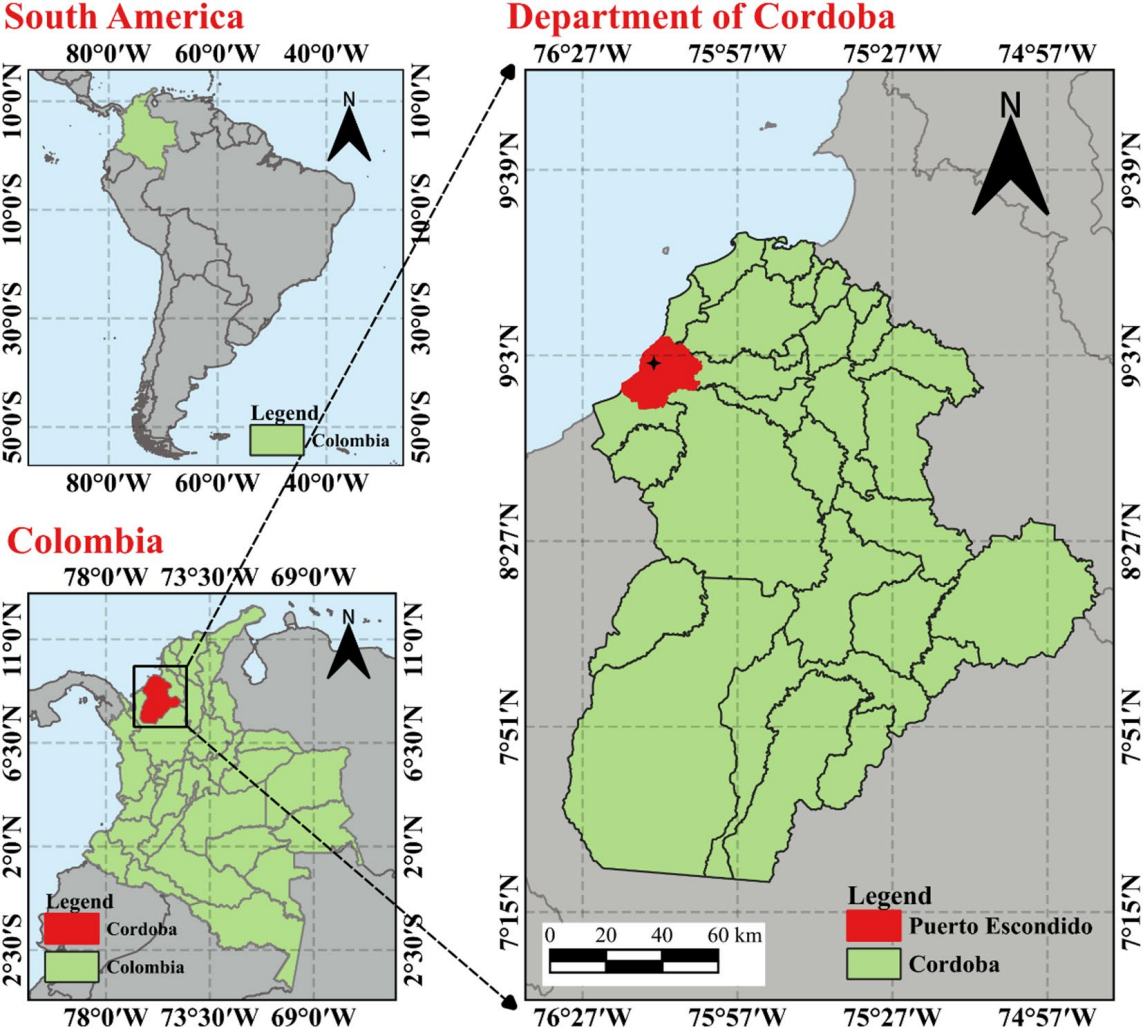
Geographic location of coconut crops in Córdoba, Colombia. This map was created through GIS information using the free QGIS software (version 3.38.2-Grenoble; http://www.qgis.org).

In countries such as Colombia, initiatives to develop new materials seek to promote the utilization of agro-industrial waste through technologies such as 3D printing^46^. This facilitates the creation of new circular economy models and eliminates linear economic models where such waste is poorly managed. Therefore, this study evaluated the effects of drying temperature, mercerizing, and epoxy coating on the physicochemical and mechanical properties of Colombian coconut fibers and their interfacial adhesion with PLA. The coir fibers used in this study were obtained from the mesocarp of *Cocos nucifera* fruit husks. This palm species grows abundantly in the coastal region of Colombia in the Caribbean. The locations of the coconut cultivation areas are shown in Fig. 1. *Cocos nucifera* belongs to the *Arecaceae* family and was first described by Carl Linnaeus in 1753^47^. The fibers were dried and then analyzed via physicochemical and mechanical characterization techniques, including optical microscopy (OM), scanning electron microscopy (SEM), Fourier transform infrared spectroscopy (FTIR), and tensile testing. Additionally, bulk density measurements were taken to calculate the density of the coconut fibers, and a bromatological analysis was performed to assess their chemical composition. Furthermore, the fibers underwent a mercerization treatment and an ER coating via an impregnation process to evaluate their interfacial performance within the PLA matrix through a single-fiber pull-out test. Finally, potential fiber‒matrix interactions were investigated via sessile drop testing, FTIR, and SEM.

## Materials and methods

### Materials

**Fig. 2:**
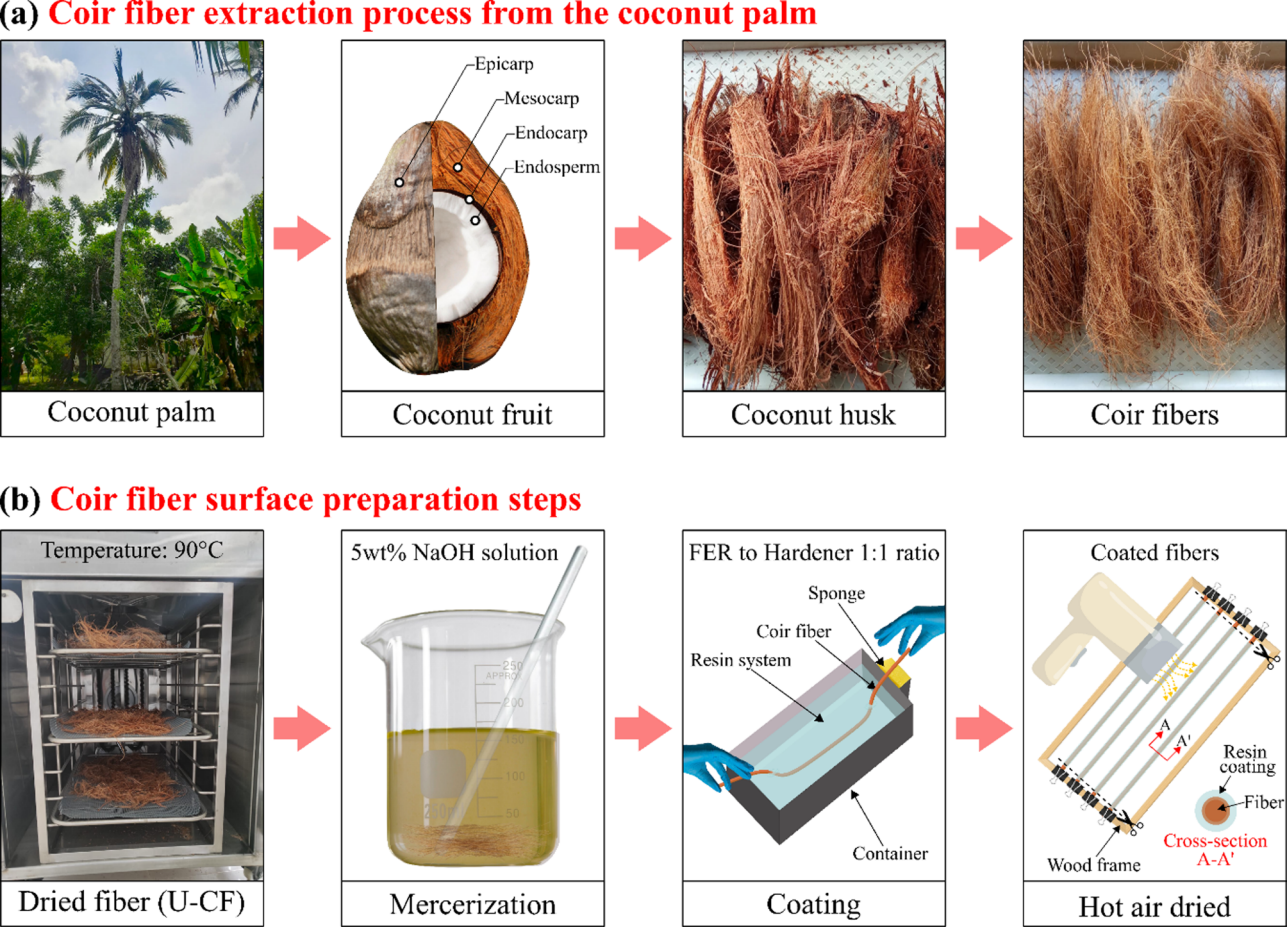
**a** Coir fiber extraction from the coconut palm tree and **b** Coir fiber surface preparation steps.

The CFs used in this study were sourced from the mesocarp of Cocos nucifera palm fruits harvested in the municipality of Puerto Escondido, Córdoba, Colombia, as indicated in Fig. 1. The geographical coordinates of the sample collection area are 9°01′09″N 76°15′41″W. The palm tree has been cataloged under the registration number COAH/55,556 by María Forero at the Colombian Amazon Herbarium (COAH) of the Amazon Institute of Scientific Research (SINCHI)^48^. A voucher sample of coconut mesocarp fiber is preserved in the herbarium of the University of Cordoba under catalog number HUC 9780. The general process for extracting CF from coconut palm is shown in Fig. 2a. After the coconut palm fruit is harvested, the coconut husk (i.e., exocarp and mesocarp) is separated from the endocarp. Next, the mesocarp was manually separated from the exocarp and then immersed in water for 48 h (retting) to soften the fibers and facilitate their extraction. Afterward, the fibers are separated manually and then washed to remove any remaining pulp or impurities. Finally, the CFs were vacuum stored in plastic bags for preservation of their extraction conditions. On the other hand, a commercial eSUN^®^ 3D-printed PLA filament with a diameter of Ø1.75 mm was purchased from Arrowti3D, Bogotá, Colombia, with a density of 1.25 g/cm³ and a printing temperature ranging from 190 to 210 °C. A flexible epoxy resin (FER) system consisting of components A and B (resin and hardener, respectively) was purchased from Carbon Fiber Stocks in Medellín, Colombia. The density of the mixture is 1.06 g/cm^3^, with a viscosity of 800–1000 mPa-s and a Shore D hardness of 30–35 (obtained from the manufacturer’s test report).

## Bromatological analysis and bulk density

Bromatological analysis was conducted on the oven-dried CF at a temperature of 90 °C. The mass percentages of acid detergent fiber (ADF), neutral detergent fiber (NDF), and cellulose were determined following the standard methods recommended by the AOAC International official method 2002.04^49^. The mass percentages of lignin and hemicellulose were calculated via Eqs. (1) and (2).

1 $${\text{Lignin}}\left( \% \right)={\text{ADF}}\left( \% \right) - {\text{Cellulose}}\left( \% \right)$$

2 $${\text{Hemicellulose}}\left( \% \right)={\text{NDF}}\left( \% \right) - {\text{ADF}}\left( \% \right)$$

In addition, the bulk density of the CFs was determined via the ISO 5311:1992 standard. A predefined volume container with internal dimensions of 508.33 cm^3^ and a calibrated weight of 339.2 g was used. The CFs dried at 90 °C were previously ground. Five different measurements were performed to obtain an average value of the apparent density of the CF. The bulk density (*ρ*_*a*_) was calculated via Eq. (3).

3 $${\rho _a}=\frac{m}{V}$$

where *m*­ is the mass of the ground fiber deposited in the container (g) and *V*­ is the internal volume container (cm^3^.

## Drying procedure

To evaluate the influence of drying temperature on the moisture content (*MC*) of CFs under extraction conditions, the fibers were dried in a BPG-9010 A Model I convection oven at temperatures of 40 °C and 90 °C. The choice of both temperatures was based on practical relevance and scientific literature. Specifically, 40 °C is close to the typical ambient temperature in many Caribbean regions. Therefore, this temperature can be considered representative of passive solar drying processes that utilize renewable resources^50^. This approximation is particularly relevant for circular economic applications in contexts with limited infrastructure. In contrast, 90 °C is close to the evaporation point of water and is usually used for drying LFs^50,51^. For each temperature, 250 g of fibers were placed on three drying trays and weighed via an Adventurer ARC120 analytical balance with an accuracy of ± 0.1 g. The mass loss was continuously measured at 10- and 20-minute intervals until two consecutive measurements showed no mass variation greater than 1%. The moisture content (MC) was calculated via Eq. (4), following the ASTM D4442-20 standard.

4 $$\:MC\left(\%\right)=\frac{A-B}{B}\times\:100\%$$

where *A* ­ represents the original mass of the CFs (g), and *B* ­ represents the oven-dried mass at time t (g). Additionally, the experimental drying curves at both temperatures were obtained by periodically sampling the moisture content^52,53^. These curves were then fitted via the diffusion approximation model described in Eq. (5), which has been considered by De Oliveira et al.^51^ and Montoya et al.^50^ as the most suitable for describing the MC curve of lignocellulosic fibers extracted from agro-industrial residues. The model parameters were adjusted through nonlinear regression via Polymath^®^ 6.10.

5 $$\:MC=a{\:e}^{-kt}+\left(1-a\right){\:e}^{-kbt}$$

## Characterization of the dried fibers

### Diameter measurement

To measure the diameter of the CFs, images of longitudinal sections of the fibers were taken via a Leica model EZ4D stereomicroscope (Wetzlar, Germany). Twenty random fibers were selected from oven-dried samples at 40 and 90 °C, and 15 transverse measurements were taken from different regions along the fiber length. The images were then processed via free ImageJ^®^ software.

### Scanning electron microscopy

The morphology of the dried CFs at 40 and 90 °C was examined via a JEOL^®^ model JSM-7100 F FEG scanning electron microscope (Tokyo, Japan) with an acceleration voltage of 5 kV and a working distance (WD) between 19 and 21 mm. The samples were fixed to brass mount holders with double-sided carbon tape and then ion-sputtered with gold via a Quorum model Q300T D sputtering system to ensure conductivity (Laughton, United Kingdom).

## Fourier transform infrared spectroscopy

The dried CFs were examined and analyzed by Fourier transform infrared spectroscopy (FTIR) on a Nicolet™ Summit spectrometer (Thermo Fisher Scientific, Waltham, USA) equipped with an Everest™ Diamond ATR (Attenuated Total Reflectance) accessory. Spectra were collected in the wavenumber range of 500–4000 cm^− 1^ with a resolution of 4 cm^− 1^. Peaks were analyzed and compared to those in open-access databases.

## Tensile test

Dried CFs at 40 and 90 °C were tensile tested according to ASTM D3822–07 using a Shimadzu^®^ model Autograph AG-X universal testing machine (Kyoto, Japan) equipped with a 500 N load cell. Single fibers were mounted in cardboard templates, and their gauge length was adjusted to 50 mm. A total of 20 samples were tensile tested for each drying temperature at a crosshead speed of 5 mm/min. The tests were conducted at a temperature and relative humidity of 25 °C and 50%, respectively. Mechanical properties such as the ultimate tensile strength (UTS), Young’s modulus, and elongation at break were evaluated. Optical microscopy images were taken for diameter measurements from transverse dimensions along the fiber, and the cross-sectional area was calculated assuming a fully circular cross-section. The samples that failed within the calibrated length were considered for calculating the mechanical properties.

### Data analysis

The tensile test results were analyzed via open-source R software (version 4.2.2, available at https://www.r-project.org/) within the RStudio development environment (version 2022.12.0.353, available at https://posit.co/download/rstudio/). Descriptive statistics and outlier checks (1.5 IQR method) were conducted for each sample. The assumptions of normality and homoscedasticity of variance were evaluated by Shapiro‒Wilk’s test and Levene’s test, with a significance level of 95%. Since the assumption of homogeneity of variance was violated (P value < 0.05), a two-tailed Welch’s t-test was performed on the mechanical properties of the dried CFs to study significant differences in the mean values of the response variables. A significant level of 0.05 was established. Null hypothesis: the means of the two groups (i.e., dried CF at 40 °C and 90 °C) are equal. Alternative hypothesis: the means of the two groups are different.

### Preparation of CF surfaces under different conditions by mercerization and polymeric coating

**Fig. 3:**
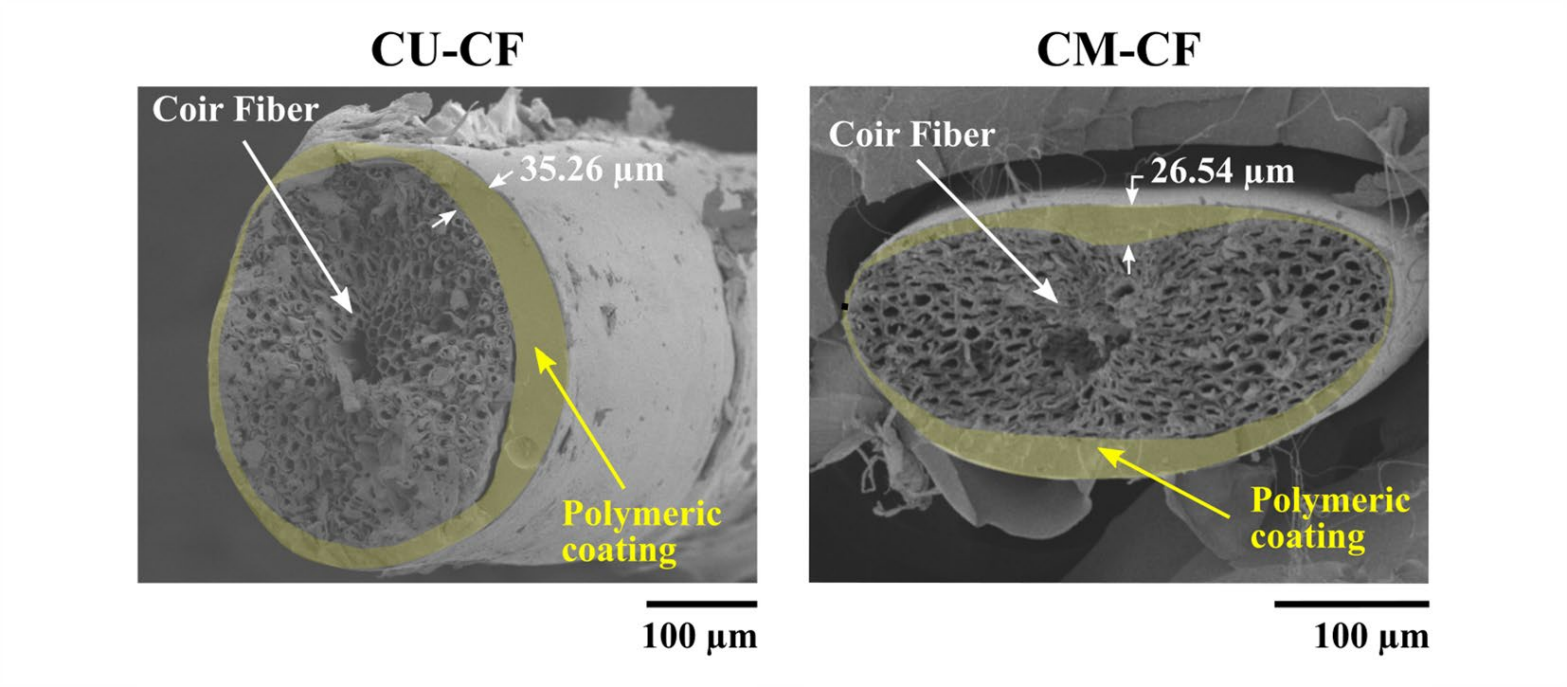
Cross-section of fibers with polymeric coating: CU-CF and CM-CF.

The CFs were prepared in four steps according to the process shown in Fig. 2b. First, the raw coconut fibers were oven-dried at 90 °C for 2 h under extraction conditions to remove moisture. These fibers are referred to as untreated coir fibers (U-CFs). Next, fibers approximately 150 mm in length were hand-selected from these samples for subsequent treatments. Second, the U-CFs were immersed in a 5 wt% NaOH solution at room temperature for 1 h with constant stirring. The solution concentration was stabilized based on findings from the literature^54^ and to minimize residual chemicals in the process effluent. The fibers were then washed with distilled water and acetic acid (50% v/v) to remove any remaining NaOH solution and dried in a controlled atmosphere at 90 °C. These samples were referred to as mercerized coir fibers (M-CFs). FER and the hardener were mixed at a 1:1 ratio, stirred, and poured into a polypropylene container. The CFs were then immersed in the resin mixture to ensure uniform impregnation, and the excess resin was removed by applying pressure to a sponge. Finally, the coated CFs were placed on a wooden frame using paper clips. A hot air blow dryer was used to accelerate the curing process. Afterward, the coated CFs remained at room temperature for 24 h. In this study, two types of coated CFs were prepared: (1) those coated without chemical treatment (CU-CF) and (2) those coated after prior mercerization (CM-CF). Figure 3 shows SEM images of the cross-section of the coated CFs. The polymer coating surrounded the fiber’s outer surface. No polymer penetration in the lumens was observed. The mean layer coating thickness obtained for both CU-CF and CM-CF via the preparation method was 25.59 ± 12.44 μm.

### Characterization testing

#### Morphological and chemical characterization

Optical microscopy (OM) images of the longitudinal sections were taken using a Leica^®^ EZ4 transmission-mode stereomicroscope. In addition, a JEOL^®^ JSM-7100 F scanning electron microscope (SEM) (Tokyo, Japan) was used to analyze the evolution of the CF morphology with alkali treatment and coating. The U-CF, M-CF, CU-CF, and CM-CF samples were examined by applying an accelerating voltage of 5 kV and a gold coating to improve the conductivity. In addition, FTIR spectroscopy was employed to identify the functional groups in the different CF and matrix samples. The measurements were performed via a Shimadzu IRTracer-100 spectrometer (Kyoto, Japan) equipped with an attenuated total reflectance (ATR) accessory, covering a spectral range of 500 cm^−1^ to 4000 cm^−1^ with a scan resolution of 4 cm^−1^.

### Contact angle measurement

**Fig. 4:**
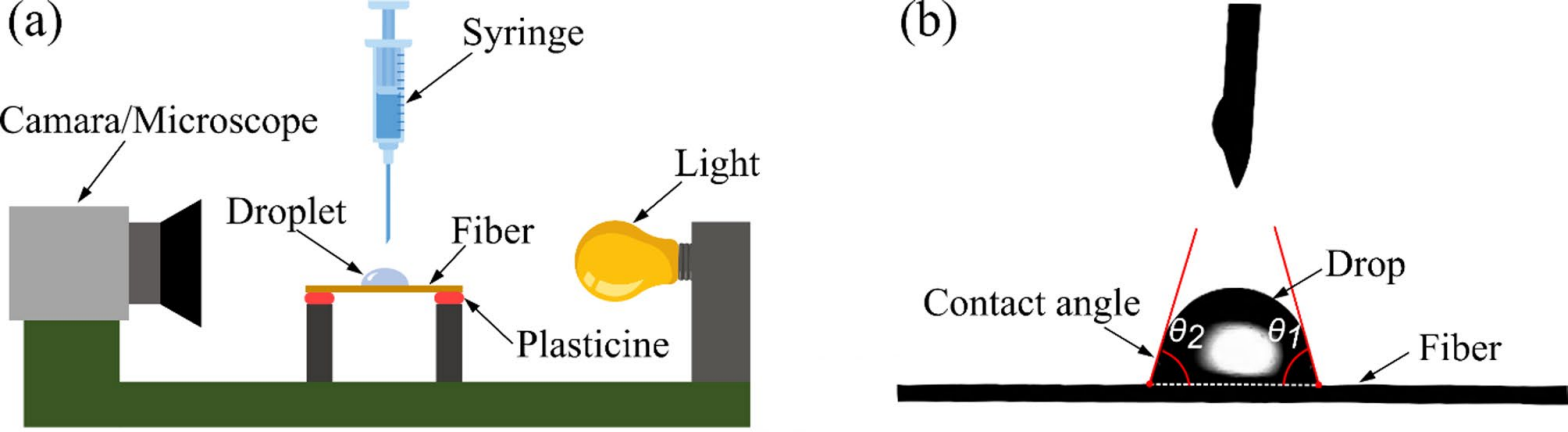
**a** Experimental setup used for CA measurement^56^ and **b** Contact angle measurement on fiber.

To evaluate the contact angle (CA) of the CFs (untreated, treated, and coated), the procedure of ASTM D7334 was followed. A drop of approximately 0.7 µL of deionized water was placed on the surfaces of the fibers to be tested. A digital microscope was used to magnify the contact area, and images were taken within 30 s of drop deposition. Figure 4a shows the experimental setup used for CA measurement. Five replicates were made for the U-CF, M-CF, CU-CF, and CM-CF conditions, and two angle measurements were taken, one at each edge of the drop (see Fig. 4b). The average of the ten measured angles was then calculated.

### Tensile test

In this work, the mechanical properties of untreated CFs (i.e., CFs dried at 90 °C) were compared with those of the M-CF and CM-CF samples. M-CF and CM-CF samples with a 50 mm gauge length were tested at a crosshead speed of 5 mm/min. Tensile properties such as the UTS and Young’s modulus were calculated via the dried CF calculation method. An analysis of variance (ANOVA) was performed to study the influence of the different surface treatments on the responses considered. Null hypothesis: The means of all groups are equal. Alternative hypothesis: the means of one or more groups differ at the confidence level of 95%. Assumptions of normality and homogeneity of variances were checked via Shapiro-Wilk and Levene’s tests with a significance level of 0.05. Since the assumption of homogeneity of variance was violated in this study when the Young’s modulus and UTS values were analyzed, one-way Welch’s ANOVA was applied, followed by pairwise comparisons via the Games–Howell test. Welch’s test performs the best in three-group heterogeneity cases when the normality assumption is met for equal or unequal sample sizes^56^. Twenty samples per group were tested, but only those that failed in the calibration gauge length were considered. Furthermore, a significance level of 0.05 was selected to evaluate the significant differences between the groups. The calculation was conducted via open-source R software.

### Single-fiber pull-out tests

Single-fiber pull-out tests were performed between the CFs (untreated, mercerized, and coated) and the two polymer matrices, FER and PLA. The samples were prepared according to the procedure described by^57^. Details on sample preparation can be found in Figs. S1 and S2, as well as Table S1 in Annexure I of the supplementary material. Table 1 shows the experimental configurations with different CF conditions and matrices. Single-fiber pull-out tests were performed on a Shimadzu^®^ model Autograph AG-X universal testing machine (Kyoto, Japan) using a 100 kN load cell, and a crosshead speed of 1 mm/min was set. Fiber diameters were measured via optical microscopy. A total of 60 samples were tested. Additionally, the IFSS was calculated via Eq. (6) by dos Santos et al.^58^. *P* represents the applied load (N), *d* represents the fiber’s equivalent diameter, and *l* represents the embedded length of the fiber within the matrix. Pull-out test results were statistically analyzed via one-way ANOVA with a confidence level of 95%. In addition, the assessed normality and homogeneity of variance were examined via the Shapiro–Wilk test and Levene’s test, with a significance level of 0.05. Analyses were conducted via open-source R software.

6 $$\:IFSS\left(MPa\right)=\frac{P}{\pi\:dl}\times\:100\%$$

**Table 1:** Experimental configurations for pull-out test.

Surface treatment	Matrix type	Nomenclature
Mercerization	FER	M-FER
Untreated		U-FER
Mercerization	PLA	M-PLA
Untreated		U-PLA
Mercerization and coated		CM-PLA
Untreated and coated		CU-PLA

U: untreated coir fibers; M: mercerized fibers; CM: mercerized and coated fibers with epoxy resin; CU: untreated and coated fibers with epoxy resin; PLA: Polylactic acid, and FER: Flexible epoxy resin.

## Results and discussion

### Bromatological analysis and bulk density results

Table 2 shows the mass percentages of ADF, NDF, cellulose, lignin, and hemicellulose obtained via bromatological analysis. The main lignocellulosic constituents in the CF were cellulose and lignin, with percentages of 34.75% and 34.01%, respectively, while hemicellulose accounted for 5.27%. The chemical composition of LFs can vary based on environmental conditions, cultivation methods, geographic location, and plant age^30,59^. Despite this variability, the values obtained in this study are consistent with those reported by other authors (see Table 3). Compared with other LFs, CFs has a higher lignin content, which makes it more moisture resistant^14^. This characteristic makes it suitable for composites owing to their potential durability. Furthermore, owing to its relatively low cellulose content and hierarchical structure, it has a relatively high elongation at break but a relatively low tensile strength^14,60^. Therefore, CF may enhance the mechanical properties of polymeric materials such as PLA, particularly in terms of impact resistance and toughness, as PLA is inherently brittle^61,62^. Similarly, the low hemicellulose content improved the thermal properties of the CFs. According to Manral et al.^63^a higher hemicellulose content in LFs reduces their thermal stability at elevated temperatures.

**Table 2:** Chemical composition of dried CF at 90 °C.

Component	Mass Percentage (%)	Used Technique
ADF	68.76	AOAC 973.18
NDF	74.03	AOAC 2002.04
Lignin	34.01	Equation (3)
Cellulose	34.75	AOAC 973.18
Hemicellulose	5.27	Equation (4)

**Table 3:** Chemical composition of CFs researched by several authors.

Origin	Cellulose (% weight)	Hemicellulose (% weight)	Lignin (% weight)	References
Jamaica	32.65	7.95	59.40	Jústiz-Smith et al.^65^
India	27.41	14.63	42	Narendar et al.^66^
Brazil	14.0–19.0	1.0–4.0	8.0–14.0	Martinelli et al.^31^
Colombia	35.99	10.51	48.94	González-Delgado et al.^67^

The bulk density of the CF was 0.65 ± 0.005 g/cm³, which was slightly higher than the range reported for Indian coconut (0.22–0.34 g/cm³) by^67,68^. This difference may be attributed to factors influencing the measurement method, including the particle size and the volume of space between the particles^69^. Nevertheless, this value is comparable to that of other fibers used to reinforce composites, such as jute, kenaf, flax, and curaua^70^. A low density is desirable for producing biobased composites, as it enables the fabrication of lightweight and biodegradable components^71^. These characteristics make CF a versatile material for reinforced polymer composite applications^19,72^.

### Drying analysis

**Fig. 5:**
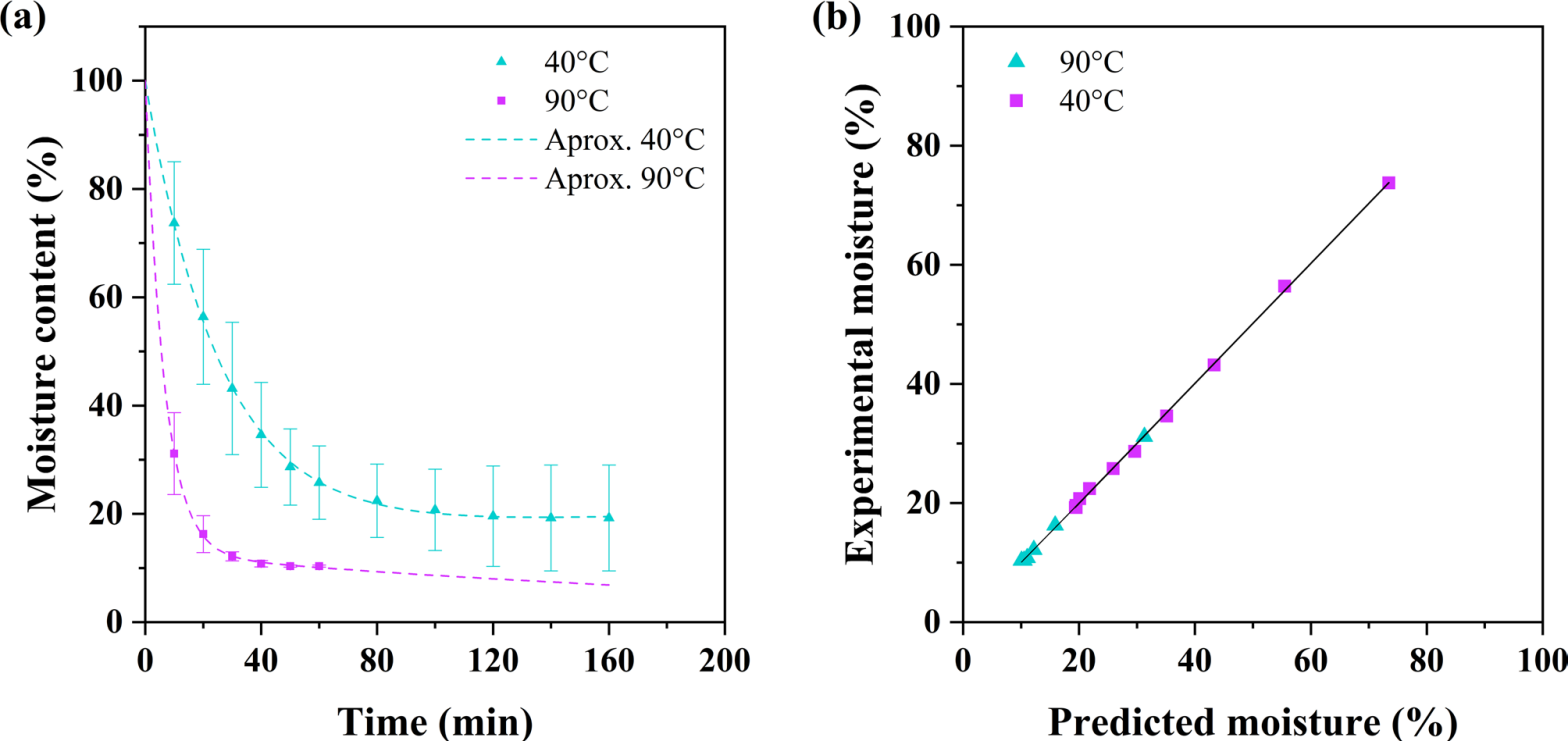
Drying curve with the diffusion approximation model fitted using experimental data.

Figure 5 shows the results of the drying curve analysis and diffusion approximation model for CF. As shown in Fig. 5a, the moisture content of the samples decreased over time. For the samples dried at 40 °C, the MC decreased to 19.2 ± 9.9% after 120 min, whereas for the samples dried at 90 °C, the equilibrium MC was 10.8 ± 0.6% after 40 min. Furthermore, the drying curve at 90 °C had a steeper slope during the initial period, reaching an MC of 16.6 ± 3.4% within the first 20 min. Since temperature is the main driving force for moisture evaporation, higher temperatures, such as 90 °C, facilitate the removal of free water from fibers because of the increased thermal energy available^51,73,74^. Consequently, the moisture removal rate is greater, resulting in lower water retention in the dried CFs at 90 °C after retting. These results are consistent with those of previous studies^51,75^which associated higher drying temperatures with increased moisture removal rates in lignocellulosic materials. Furthermore, the good fit of the model to the experimental data indicates that the drying kinetics of the studied CFs are well described by the diffusion approximation model (Fig. 5a). Table 4 summarizes the fitted model parameters and the corresponding statistical analysis results. The adjusted R² values approached 1, whereas the ERMS and χ² values were near zero for both drying temperatures, which further supports that the model appropriately represents the drying curve of this lignocellulosic material. Additionally, Fig. 3b reveals a 45° slope between the experimental moisture content and predicted values, validating the diffusion approximation. This finding agrees with the results reported in a previous study^50^.

**Table 4:** Results of parameters and regression coefficients for diffusion approximation model.

Drying temperature	a	k	b	*R* ^2^	*R* ^2^_adj_	ERMS	χ^2^
40 °C	0.8309	0.0387	-0.0216	0.9996	0.9995	0.0014	3.32 × 10^− 5^
90 °C	0.8733	0.1521	0.0251	1.0000	0.9999	0.0008	8.10 × 10^− 6^

### Effect of drying temperature on the properties of Coir fibers

#### Fiber diameter analysis

**Fig. 6:**
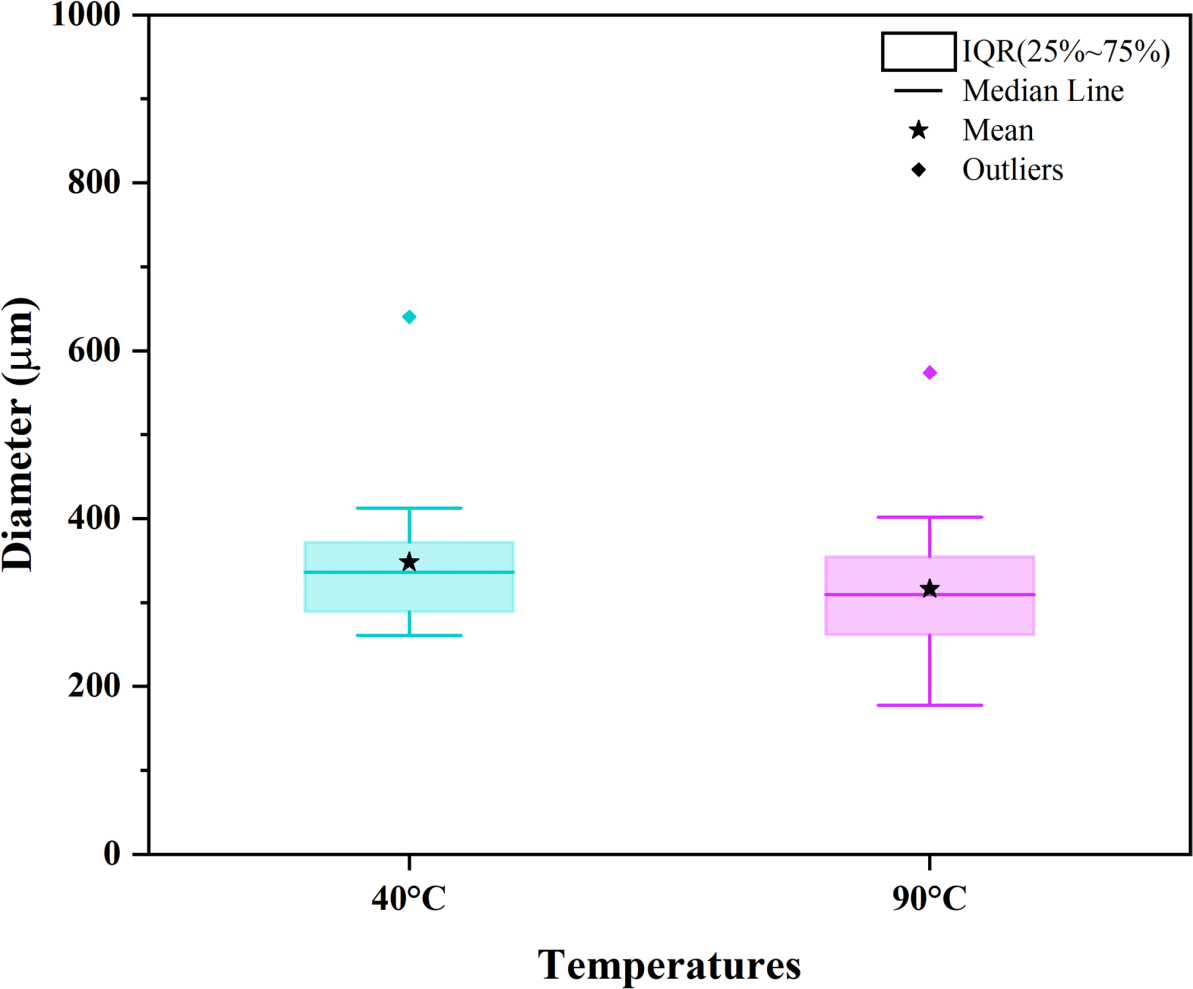
Diameter distribution of dried CFs at 40 °C and 90 °C.

Figure 6 shows the results of the CF diameter analysis via a box‒whisker plot. The mean diameter decreased slightly with increasing temperature. The samples dried at 40 °C had a mean fiber diameter of 348.06 ± 82.19 μm, whereas the samples dried at 90 °C had a mean fiber diameter of 316.00 ± 86.42 μm. However, the median of the 90 °C sample falls within the interquartile range (IQR) of the 40 °C sample. This finding indicates that the central tendency of the 90 °C sample is within the range of values of the 40 °C sample. Compared with the 40 °C sample, the 90 °C sample presented a longer box-whisker plot, indicating greater data variability with smaller diameter values. Furthermore, the shape of the box plots suggests that the fiber diameters in both groups have a symmetrical distribution. Nevertheless, outliers in both samples were observed, which may result from the heterogeneous characteristics of LFs^76^. The CF diameters reported in this work are consistent with those reported in the literature^77^. The decrease in the CF mean diameter may be associated with the increase in drying temperature. Authors such as Martinelli et al.^30^ have attributed this behavior to the diametrical shrinkage of the fibers due to moisture loss. Drying at 90 °C resulted in a greater reduction in the MC, which explains the observed slight decrease in fiber diameter in the 90 °C samples.

#### Morphological analysis

**Fig. 7:**
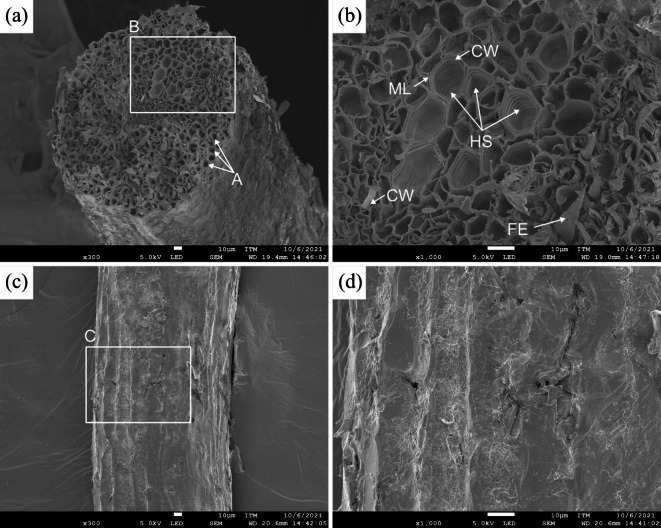
SEM images of dried CF at 40 °C: **a** cross-section, **b** region B of **a** magnified to 1000$\:\times\:$, **c** longitudinal section, and **d** region C of **c** magnified to 1000$\:\times\:$.

Figure 7 shows SEM images of the cross-sectional and longitudinal sections of the CF at 40 °C. The CF is composed of many tubular fibers (A) with large holes, called elementary fibers, which are aligned in parallel, as shown in Fig. 7a^60^. The holes in the cross section exhibit a variety of shapes, including polygonal and suborbicular shapes, as well as varying diameters^60^. These features reflect the heterogeneity of the internal structure of the fibers. Its structure has greater porosity than other natural fibers. This porosity favors the buoyancy of coconut fruits and facilitates their dispersal in tropical and subtropical regions^14^. The magnified region (B) in Fig. 7b reveals the structural arrangement of the CF. The tubular fibers consist of a cell wall (CW) that surrounds the central lumen. These fibers are held together by the middle lamella (ML), a cementing layer primarily composed of pectin^78,79^. Additionally, the tubular fibers exhibit helical structures (HSs), as observed via SEM, which are composed of fibrils cohered with a noncellulosic matrix that is helically stacked^50,60,80^. According to the literature, these HSs are composed mainly of cellulose microfibrils^60^. This helical arrangement enhances the compressive strength and elasticity of the CF^60,80^. Furthermore, some free ends of the tubular fiber (FE) were observed at the fractured ends of the CF cross-section, which was likely caused by pullout during sample preparation. However, this observation is consistent with findings reported in the literature^60^. Figure 7c shows the longitudinal section of the CF. The outer surface of the fiber is covered by a layer composed mainly of non-cellulosic substances, such as lignin, pectin, and impurities^60,80^. These components contribute to the roughness of the fiber surface^80^. The magnified region (C) in Fig. 7d reveals that the surface is coated with a layer of impurities, which appear as flaky structures that cover the entire surface uniformly^81^. Therefore, it can weaken the interfacial bonding ability between the fiber and the polymeric matrix, causing debonding^81^.

**Fig. 8:**
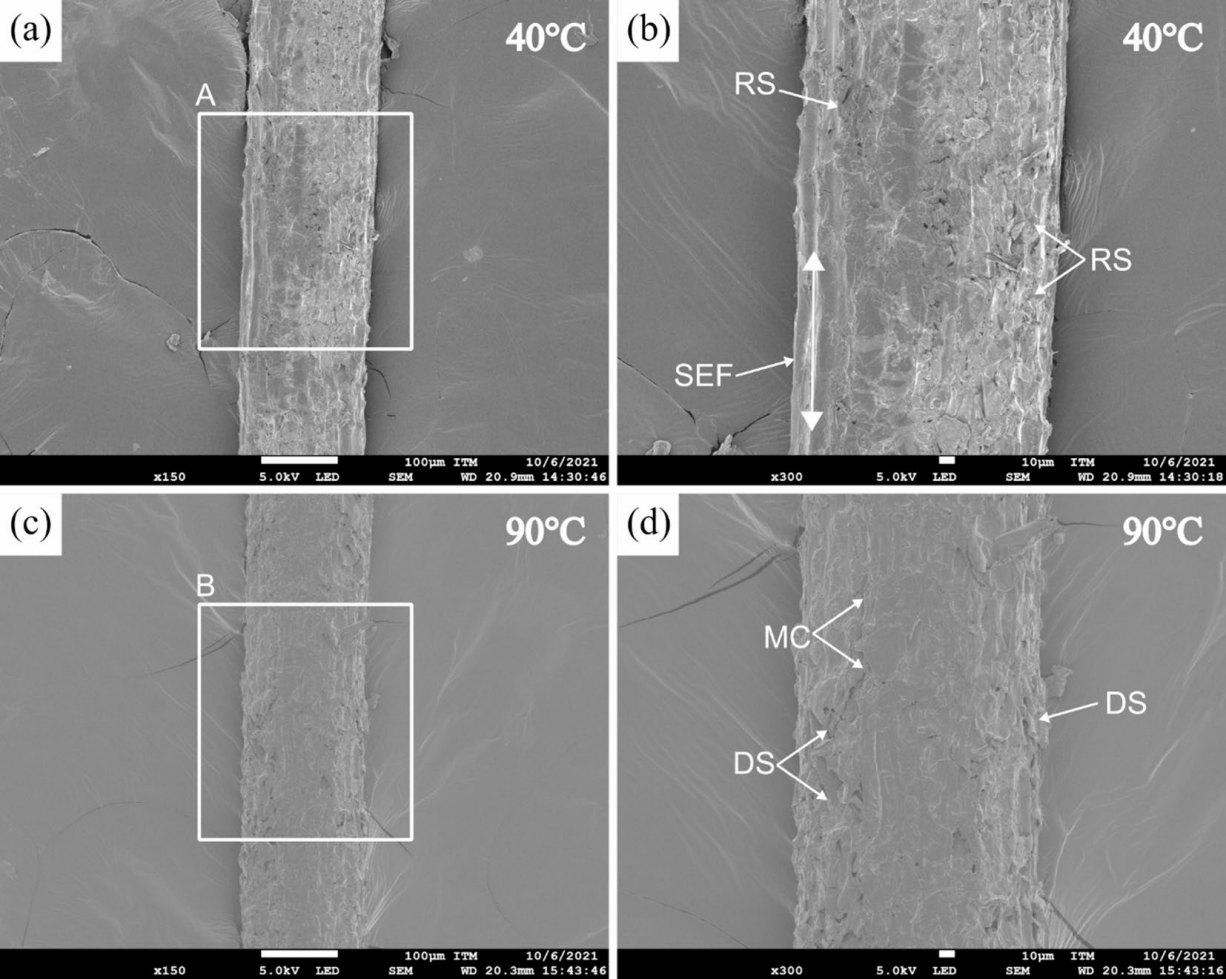
SEM images of the longitudinal section of dried CF at different drying temperatures: **a** 40 °C, **b** region A of **a** magnified to 300$\:\times\:$, **c** 90 °C, and **d** region B of **c** magnified to 300$\:\times\:$.

The SEM images in Fig. 8 show the surface morphologies of dried fibers. Figure 8a displays the longitudinal section of the dried CF at 40 °C. As previously mentioned, this sample had an outer surface with flake-like impurities uniformly covering it. The magnified image of region A (Fig. 8b) revealed a partially rough surface (RS) with strands of elementary fibers (SEF) aligned along the fiber’s longitudinal direction. The fibers dried at 40 °C presented greater cohesion, likely because this drying temperature did not induce degradation between the cellular components. In contrast, the fibers dried at 90 °C displayed irregularities, indicating that their morphology may change due to moisture loss and volatiles during drying^52^ (see Fig. 8c). Additionally, the magnified image (Fig. 8d) reveals the presence of microcracks (MCs) and a partially degraded surface (DS). These defects can be attributed to the loss of cohesion on the surface associated with the partial degradation of non-cellulosic substances^82^.

### FTIR analysis

**Fig. 9:**
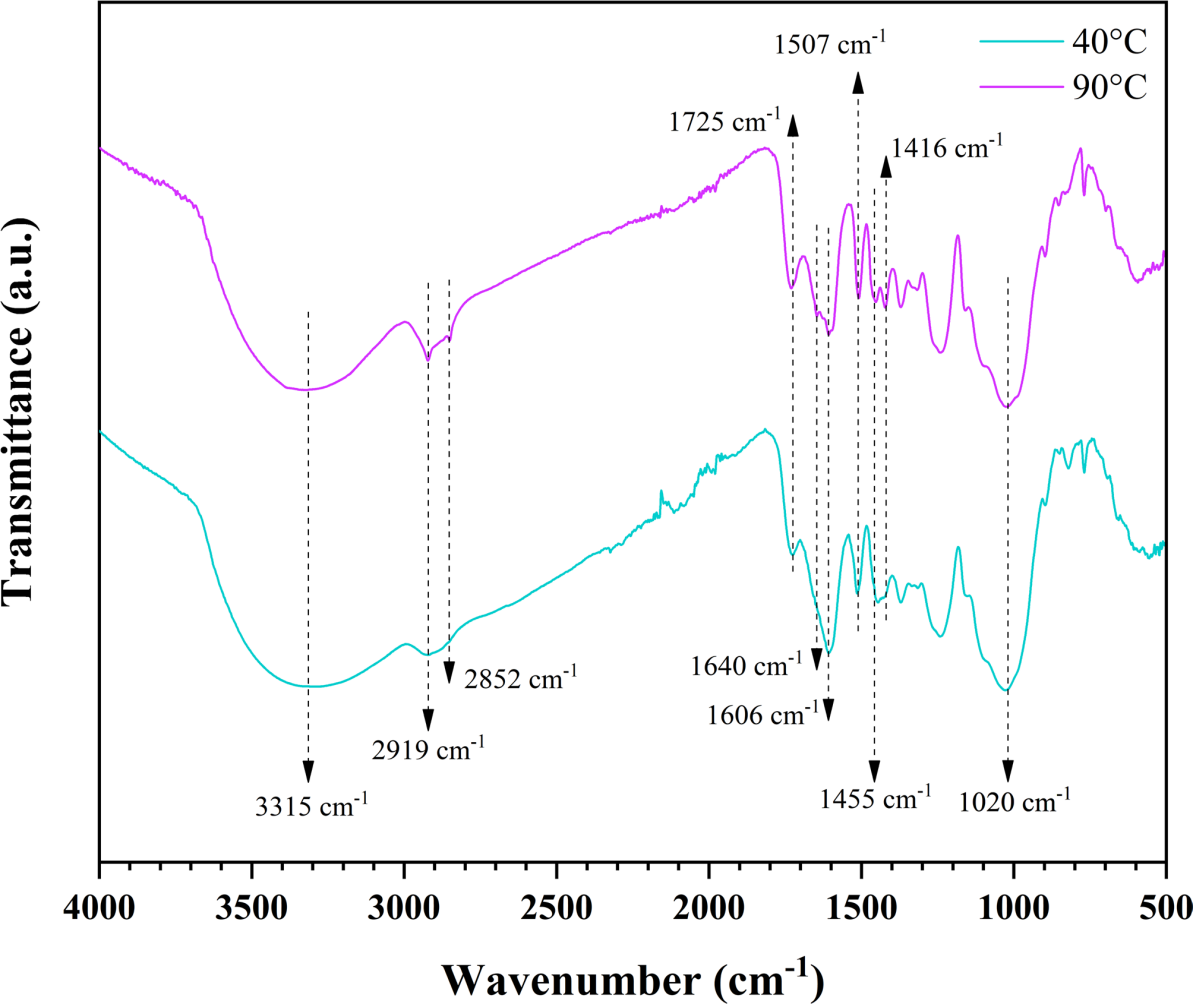
FTIR spectra of dried CF at 40 °C and 90 °C.

Figure 9 shows the FTIR spectra of the dried samples. Absorption bands associated with the lignocellulosic components of the coir fibers can be observed in the 3500 cm^−1^−1000 cm^−1^ region. Table 5 summarizes the main functional groups observed in the CFs. There were no notable differences between the two samples’ FTIR spectra, such as displacement or disappearance of absorption bands, although there were slight variations in band intensities. This may reflect possible changes in chemical composition influenced by drying temperature. For example, the intensity of the OH group associated with the absorption band at 1640 cm^−1^ decreased in the sample dried at 90 °C. This could be attributed to the greater moisture removal from the fiber during the higher-temperature drying process. Similarly, a decrease in the peaks at 1604 cm^−1^, 1455 cm^−1^, and 1416 cm^−1^ was observed, which indicates the possible removal of extractives and lignin. Furthermore, a peak was observed at 2919 cm^−1^, corresponding to the symmetrical stretching of C–H bonds associated with methylene and methyl groups. This band indicates the presence of aliphatic compounds from hemicellulose and cellulose in fibers after drying at relatively high temperatures^83^. Some works have reported that heat treatment facilitates the volatilization of low-molecular-weight components in CF^31^. Consequently, the appearance of this absorption band is likely attributed to the removal of fiber degradation products^82^. Therefore, the results suggest that a temperature of 90 °C could promote a change in the chemical composition of CF. A similar trend was reported in a previous study on banana fibers^51^.

**Table 5:** Main functional groups of the FTIR spectra of dried CF at 40 °C and 90 °C.

Wavenumber (cm^−1^)	Functional group	Assignment	References
3315	–OH	Stretching vibration of hydroxyl groups from cellulose, hemicellulose, and lignin.	Bhunia et al.^84^
3000 − 2850	C–H	Stretching vibrations of methylene and methyl groups from cellulose, hemicellulose, and lignin.	Wang et al.^15^
1725	C = O	Stretching vibration of the acetyl group in hemicelluloses.	Freitas et al.^83^
1640	Fiber–OH	O–H bending by the presence of humidity.	Horikawa et al.^80^
1606 and 1507	C = C	Stretching vibration of the aromatic skeletal of the lignin.	Wasti et al.^85^
1455	C–H	C–H deformations of lignin and C–H bending of hemicellulose.	Wasti et al.^85^ ; Paz et al.^87^
1416	C–H	C–H groups deformation from lignin and C–H group symmetric bending from cellulose.	Wasti et al.^85^
1020	C–O	Stretching vibration of C–O-C groups from cellulose and hemicellulose.	Vieira et al.^88^

#### Analysis of the tensile properties of dried Coir fibers

**Fig. 10:**
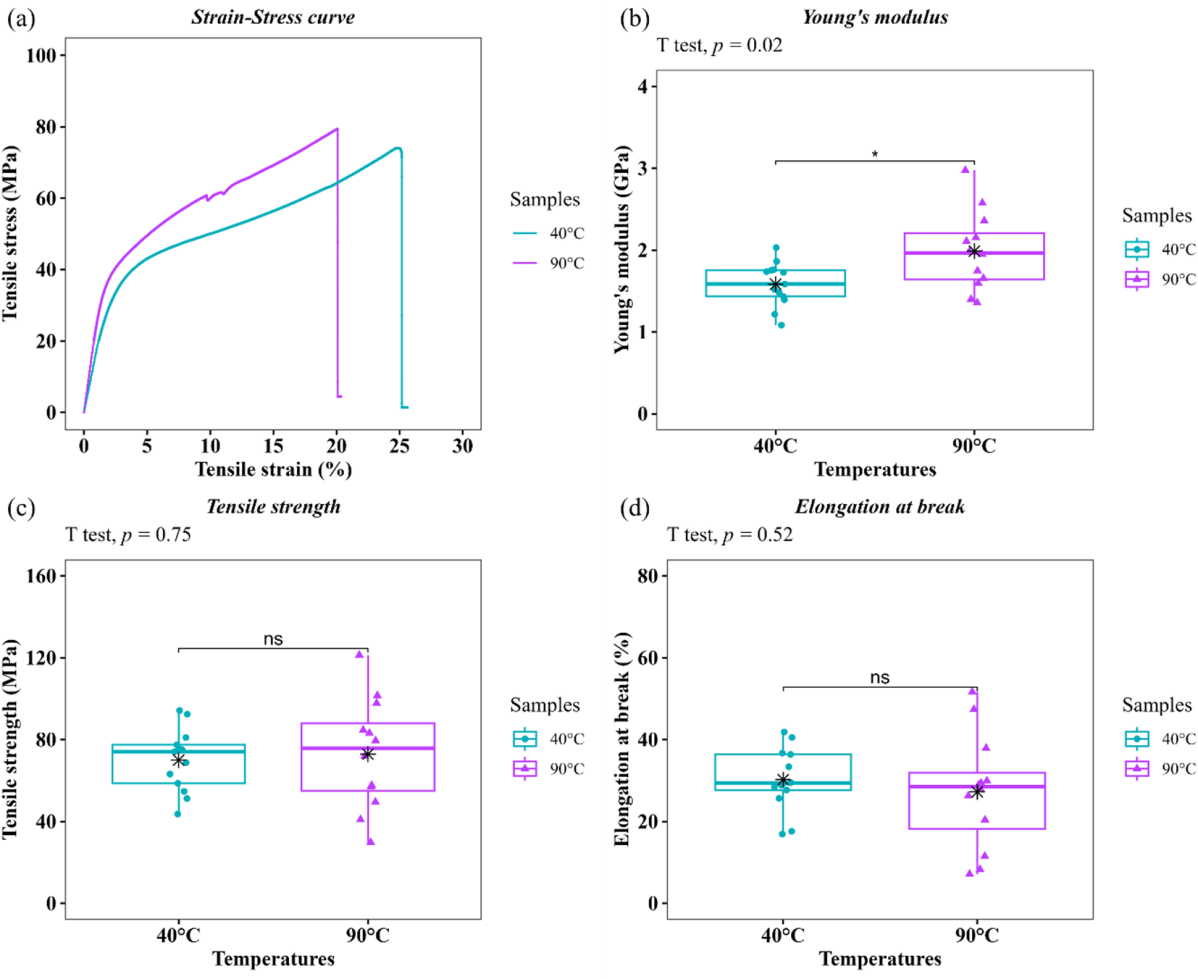
**a** Tensile stress-strain curve of dried CF at 40 °C and 90 °C. Box plots showing **b** Young’s modulus, **c** tensile strength, and **d** elongation at break of dried CF, together with the results of Welch’s t test. The black star-shaped symbol represents the mean of each Box plot. No significant difference (ns) or significant difference at a 95% confidence level (*), *p*: *P* value. Using R (version 4.2.2. https://www.r-project.org/).

Figure 10 shows the results of the analysis of the tensile properties of two dried samples. The results are also summarized in Table 6. In this study, a total of 15 and 13 samples failed within the calibrated length for each drying temperature, i.e., 40 °C and 90 °C, respectively. However, three samples were identified as outliers and removed via the 1.5 IQR method. As a result, the mean values and standard deviations were calculated using 13 and 12 samples for each group, respectively. Figure 10a shows a typical stress‒strain curve of a single tested fiber. The curves exhibit three distinct regions: an initial linear region, a nonlinear segment, and a final rectilinear ascending region, suggesting strain hardening^15,31^. This behavior is likely attributed to the hierarchical structure of the fibers, which significantly affects their mechanical response^89^. The same trend has been reported previously^2,31,90^. The mean values of both the UTS and Young’s modulus of the dried CF at 90 °C were 72.85 ± 26.94 MPa and 1.99 ± 0.48 GPa, respectively, whereas those of the dried CF at 40 °C were 69.96 ± 15.27 MPa and 1.58 ± 0.27 GPa, respectively (see Table 6). These results show a 4% increase in mean UTS and a 26% increase in mean Young’s modulus at 90 °C compared to 40 °C. The results for the UTS and modulus in this study are consistent with those reported in the literature^15,91^. At higher drying temperatures, there was a slight increase in the mean values of both the UTS and modulus, although these values exhibited greater variability (see Figs. 10b and c). In contrast, elongation at break (*e*_*b*_) decreased with increasing drying temperature. The *e*_*b*_ for CF dried at 90 °C was 27.22 ± 14.11%, whereas it was 30.21 ± 7.65% for the 40 °C samples. Despite this decrease, CF has a greater *e*_*b*_ than other lignocellulosic fibers due to its highest lignin content and the helical arrangement of its elementary fibers, characterized by a microfibrillar angle (MFA) that is not completely parallel to the direction of the fiber^15,92^. This feature enhances its suitability as a reinforcing material for brittle polymer matrices^25^. However, Fig. 10d reveals greater variability in *e*_*b*_ for the 90 °C sample, as evidenced by the longer box and whiskers. This variability could be related to the thermal degradation that the CF undergoes at 90 °C. Since the removal of degradation products leads to the formation of defects on the fiber surface, the mechanical properties can be significantly affected. The formation of microcracks and irregularities in the outer layer of the fiber reduces its ability to strain^82^. This may weaken the fiber structure and influence its mechanical response. These results are consistent with those observed via SEM.

**Table 6:** Tensile properties of dried CF at 40 °C and 90 °C.

Drying temperature	Young’s modulus (GPa)	Tensile strength (MPa)	Elongation at break (%)
40 °C	1.58 ± 0.27	69.96 ± 15.27	30.21 ± 7.65
90 °C	1.99 ± 0.48	72.85 ± 26.94	27.22 ± 14.11

The statistical results of the tensile properties of the CFs are shown in Table 7. The Shapiro‒Wilk test yielded *P* values greater than 0.05 for both samples, indicating that the normality assumption was met for the three variables studied. Additionally, although the dried CF at 90 °C showed greater variability, Levene’s test yielded *P* values slightly above the significance level (0.05). As a result, it can be assumed that the variance of each variable is equal in the two groups. Nevertheless, the Welch’s t-test was employed instead of the Student’s t-test, given that Levene’s *P* values for Young’s modulus and tensile strength approached 0.05. This approach ensures the stability of type I error rates in situations involving unequal sample sizes or heterogeneity^93,94^. According to Welch’s t-test, the difference between drying temperatures was statistically significant only for the Young’s modulus (*P* value < 0.05). Therefore, drying at 90 °C enhanced CF stiffness compared to 40 °C. The Young’s modulus increased by ~ 26% compared to the mean value of the lowest temperature. The statistical analysis can also be observed in Figs. 10b, c, and d. This result aligns with findings reported in the literature, highlighting the influence of drying temperature on CF stiffness^31,82^. The cellulose content influences mainly the Young’s modulus^89^. Since the drying temperatures used are lower than the degradation temperature of this fiber constituent^95^they are unlikely to influence the observed difference. Nevertheless, this behavior is believed to be due to changes in moisture content and possible state transitions in the fiber that occur during the drying process^96^. By increasing the drying temperature, moisture loss causes a transition from ductile to brittle, usually resulting in increased CF stiffness^31^. This shift may be associated with modifications in the hydrogen bonding network within the fiber’s microstructure. At 90 °C, water molecules are removed from between cellulose microfibrils, causing the polysaccharide chains to pack more tightly and form a denser fibrillar structure. Consequently, a more compact arrangement of microfibrils within the cell wall can contribute to an increase in the fiber’s stiffness^52,97^. Additionally, it can be that the effect of temperature on stiffness appears more pronounced in CF than in other LFs, likely due to its higher absorption of free water during extraction (e.g., retting). CFs retain more water than other LFs do because of their porous structure^31^. This may also explain the slight increase in stiffness observed when water is removed at 90 °C. Other LFs, such as banana fibers, did not show a variation in Young’s modulus with temperature^51^ On the other hand, although temperature did not affect the elongation at break or the UTS, an increase in ductility variability is associated with thermal degradation-induced defects. As previously mentioned, drying at 90 °C partially degrades the fiber’s surface, forming irregularities such as microcracks. This process involves the degradation of products and moisture loss. Therefore, it is believed that these defects act as stress concentrations, reducing the fiber’s ability to deform plastically before fracturing. Thus, implementing high drying temperatures can lead to inconsistencies in CF’s mechanical responses. Though the variation in drying temperature did not significantly affect the ultimate tensile strength or elongation at break, stiffness increased by 26% for the sample dried at 90 °C compared to the sample dried at 40 °C. This result is particularly relevant for applications that prioritize rigidity despite the slight reduction in ductility. For instance, authors such as Dong et al.^25^ have reported that the tensile modulus of PLA/CF biocomposite can increase by 25.6% with 5% wt fiber content versus neat PLA. Therefore, oven drying at 90 °C may be a better pretreatment option in terms of stiffness. As a result, the optimal drying temperature for raw CFs in this study was 90 °C.

**Table 7:** Statistical tests for tensile properties of dried CF. A *P* value$\:>$α in each test indicates no significant difference.

Statistical tests (Data group)	Young’s modulus		Tensile strength		Elongation at break	
α = 0.05	Statistics	*P* value	Statistics	*P* value	Statistics	*P* value
Shapiro-Wilk’s test (40 °C)	0.975	0.942	0.969	0.881	0.943	0.497
Shapiro-Wilk’s test (90 °C)	0.959	0.774	0.982	0.991	0.941	0.515
Levene’s test (40–90 °C)	3.07	0.093	3.87	0.0612	2.54	0.125
Welch’s t test (40–90 °C)	-2.62	0.020*	-0.327	0.748	0.650	0.524

The symbol (*) indicates a significant difference at a significance level of 95%.

#### Effects of mercerization and epoxy coating on Coir fiber–PLA interfacial adhesion

##### Scanning electron microscopy (SEM)

**Fig. 11:**
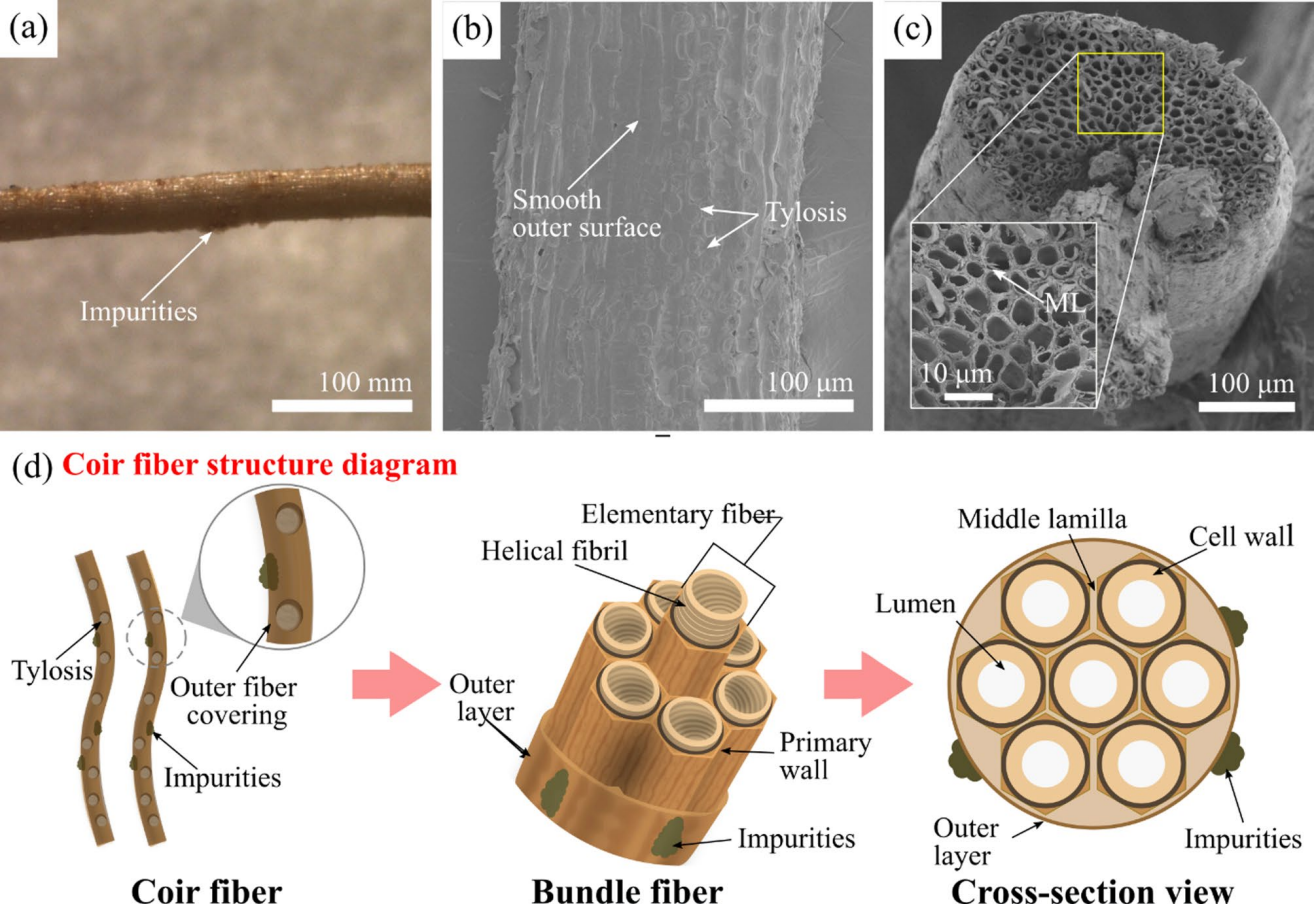
**a** Schematic representations of the coir fiber structure, **b** Optical micrograph of the longitudinal section of CF, SEM images of the U-CF: **c** Cross-sectional, and **d** longitudinal section.

**Fig. 12:**
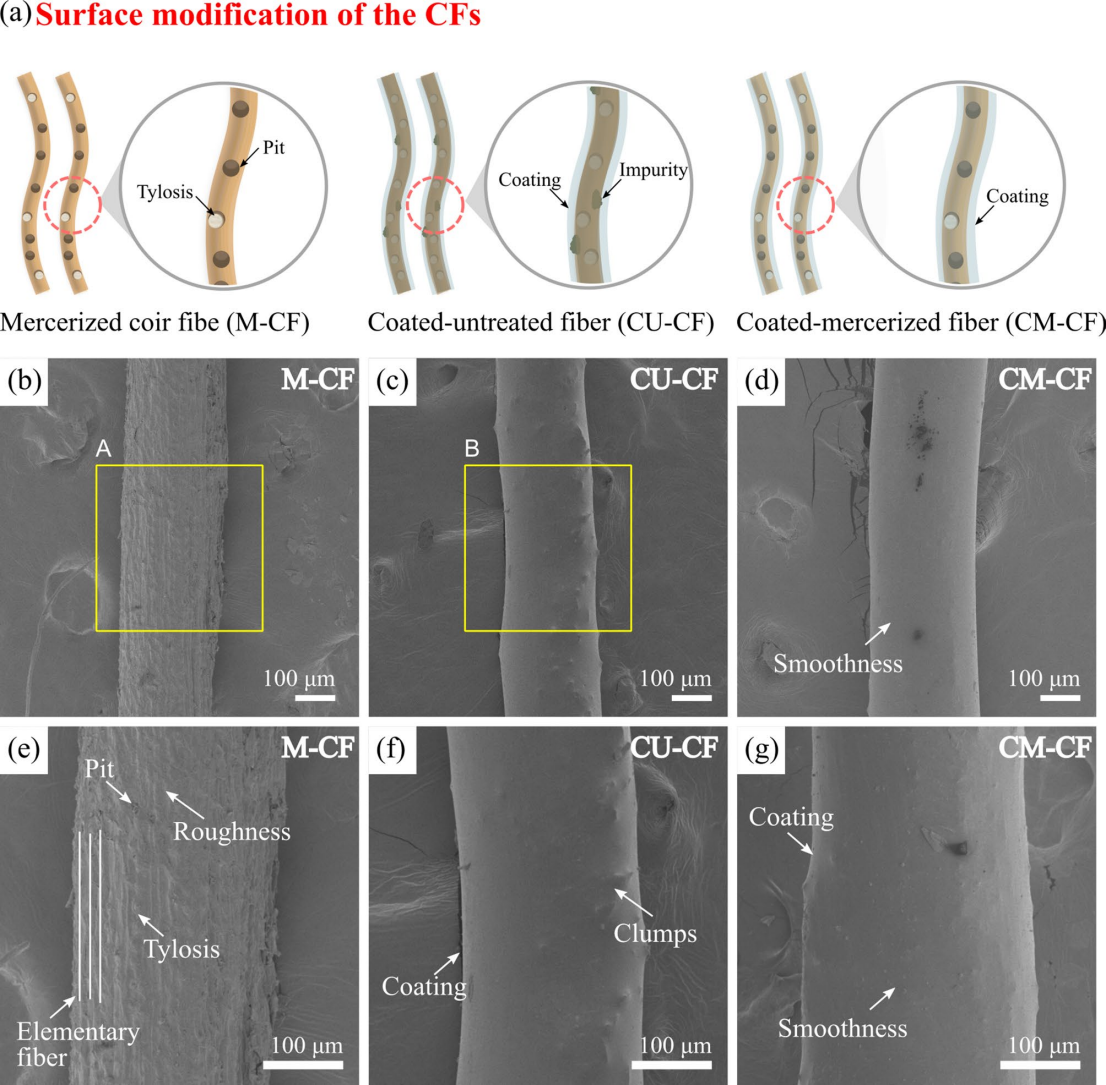
**a** Schematic diagram of the surface modification of the CFs.SEM images of the longitudinal section of CFs: **b** M-CF, **c** CU-CF, and **d** CM-CF. Images magnified to 200$\:\times\:$: **e** region A of **b**, **f** region B of **c**, and CM-CF.

The surface of LF exhibits topographical variations that can affect its mechanical interlocking with polymer matrices, resulting in a weak fiber‒matrix interface^86^. Hence, enhancing the morphology of a CF is essential for improving fiber‒matrix adhesion. In this study, OM and SEM techniques were used to analyze the surface characteristics of U-CFs. OM images revealed the presence of impurities on the fiber surface (see Fig. 11a). These impurities are believed to be remnants of coir pith, a material that remains after CF extraction and drying^98^. SEM images of the longitudinal section revealed a smooth surface with light striations, reflecting the internal structure of the fiber (see Fig. 11b). This is attributed to the presence of a layer rich in lignin and waxes that coats the surface structure^28,99^. Furthermore, tylosis was observed on the fiber surface^100^. SEM cross-sectional analysis revealed a porous structure with grouped helical fibrils, as described in the subsection “Morphological analysis” (see Fig. 11c). Elementary fibers are axially arranged and held together by the middle lamella, which is composed primarily of pectin^80^. Based on these results, a model of the U-CF structure was proposed, showing the microstructural arrangement and hierarchical structure of the CF, as shown in Fig. 11d. The U-CF surface exhibited morphological characteristics that may hinder fiber‒matrix bonding. In terms of fiber morphology, the surface contains impurities and a non-cellulosic outer layer (OL). The presence of tyloses is also evident; however, they are few visible due to this layer. At the microscale, the elementary fibers form a fiber bundle, which is covered by an outer layer. This consists mainly of amorphous materials such as lignin and long-chain fatty acids, which act as a physical barrier between the fiber microstructure and the environment^100^. However, the chemical nature of the fiber’s outer surface may be incompatible with certain polymeric matrices, limiting effective interfacial bonding. Therefore, partial removal of this layer is necessary to achieve strong interfacial bonding^32^. U-CF exhibited morphological characteristics that have the potential to influence its compatibility with the PLA matrix. Consequently, a morphological analysis was carried out on the effects of alkaline treatment and the FER coating on the CF surface. Figure 12a shows how the CF surface was modified by combining chemical treatment and a polymeric coating. Figure 12b presents an SEM image of the M-CF. The initial smooth surface of the U-CF underwent modification due to NaOH treatment. The magnified image of region A reveals an increase in the surface roughness of M-CF, which could be related to the removal of the outer layer of the fiber and impurities on its surface (see Fig. 12e)^100^. The reaction between the NaOH solution and hydroxyl groups in noncellulosic components promotes the dissolution of lignin, hemicellulose, and certain impurities, resulting in a cleaner and more uniform surface^101,102^. The removal of the layer also exposed some tyloses and pits on the surface. Based on the above model (Fig. 11d), it is believed that the chemical treatment mainly degraded the outer lignin layer and caused slight disintegration of the pectin-rich middle lamella. As a result, there was no defibrillation effect. This observation is consistent with the literature^103^. The effectiveness of a NaOH solution depends on its concentration, soaking time, and temperature^104^. These morphological changes can lead to increased mechanical interlocking between the fiber and polymer matrices, improving interfacial adhesion^86^. Figures 12c and d present SEM images of the CU-CF and CM-CF samples, respectively. It is evident that a new layer forms on the CF surface due to the polymeric coating, both for untreated and mercerized fibers^105^. The resin conformed to fiber morphology, allowing it to cover both imperfections and surface irregularities. However, irregularities in the CU-CF surface were noticed because of some clumps of REF (see Fig. 12f). This can be attributed to the presence of impurities on the untreated fiber surface, which likely hinder the wetting of the CF surface by the resin^86^. In contrast, Fig. 12g shows that the CM-CF surface looked smooth. After the treated fiber was coated with polymer, the pits and tyloses that resulted from the removal of the outer CF layer due to the chemical treatment were not observed. The epoxy resin coating filled the fiber irregularities. The coating can help maintain the fiber’s structural integrity by preventing irregularities from becoming starting points for mechanical failure.

### Fourier transform infrared spectroscopy (FTIR)

**Fig. 13:**
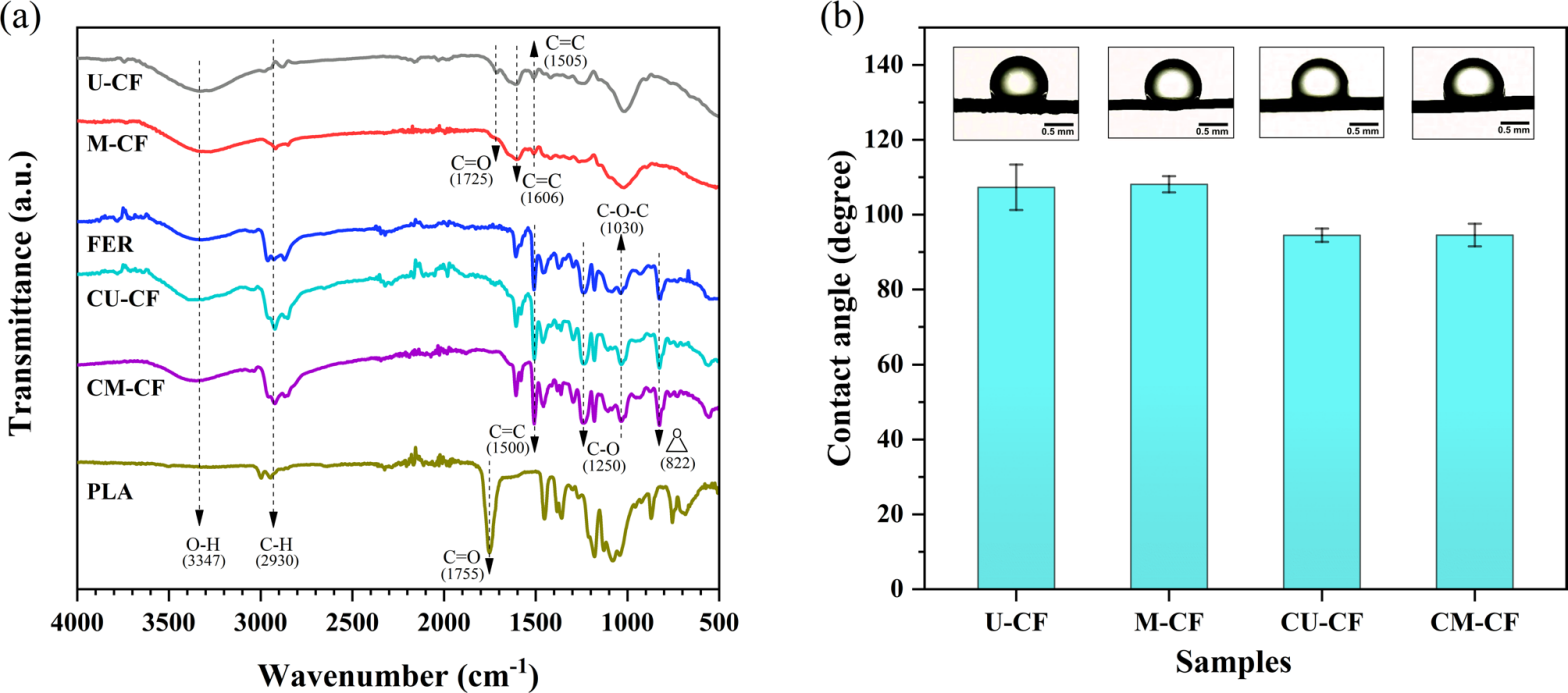
**a** FTIR spectra of untreated coir fiber (U-CF), fibers treated with NaOH solution (M-CF), coating agent (FER), untreated and coated fibers (CU-CF), coated and mercerized fibers (CM-CF), and PLA matrix. **b** Measured contact angles (°) of U-CF, M-CF, CU-CF, and CM-CF. The liquid droplet used was deionized water.

The FTIR technique was employed to investigate the variations in the functional groups of the different samples, as well as potential chemical interactions with the polymer matrices. Figure 13a displays the FTIR spectra of PLA, FER, CM-CF, CU-CF, M-CF, and U-CF. For U-CF, several absorption bands characteristic of LFs were observed. Table 5 in the subsection “FTIR analysis” contains a prior description of these bands. Compared with the U-CF samples, the M-CF samples presented a decrease in the intensity of several absorption bands following alkali treatment. A slight decrease in the band intensity at approximately 3347 cm^−1^ was observed, which may be attributed to the disruption of O-H groups due to the removal of lignin^106^. The absorption band at 1725 cm^−1^, corresponding to the C = O stretching vibration in the acetyl group of hemicellulose, nearly disappeared. This finding indicates that partial hemicellulose degradation may also have occurred during the mercerization treatment^107^. Additionally, a decrease was observed in the bands at approximately 1606 cm^−1^ and 1507 cm^−1^. This is attributed to the partial removal of lignin^86,92,108^. There was no significant change in the characteristic absorption bands of cellulose after mercerization, indicating a minimal effect on this component^92^. These results are consistent with the SEM analysis, suggesting the likely removal of noncellulosic material from the fiber surface after chemical treatment. The FER spectrum exhibited a broad band at 3300 cm^− 1,^ which corresponds to the presence of OH groups formed by opening the epoxy ring due to curing^109^. The spectrum further revealed prominent absorption bands between 2800 and 2990 cm^−1^ associated with C − H vibrational modes^110^. A band at 1500 cm^−1^ was observed, attributed to the C = C stretching vibration in the aromatic rings of FER. Moreover, the bands at 1250 cm^−1^ and 1030 cm^−1^ were associated with asymmetric aromatic C–O stretching and the presence of ether groups^110,111^. Additionally, a band at 822 cm^−1^ was related to C − O deformation corresponding to epoxy ring vibrations^110,112^. For the coated samples, the FTIR spectra are dominated by the absorption bands of the FER. This finding suggests that the coating facilitated the chemical modification of the fiber surface. However, changes in band intensity were observed for CU-CF and CM-CF concerning FER. Owing to the lack of precise information, establishing chemical interactions between fibers and polymeric matrices can be difficult. Nevertheless, this study proposes several assumptions. During curing, the reactive functional groups of FER may react with the O − H groups of uncoated fibers, forming chemical linkages^113^. In the case of CM-CF, the partial removal of the outer layer of the fiber through mercerization treatment increases cellulose exposure on the fiber surface, creating additional reactive sites^114^. This allows the hydroxyl groups of cellulose to react with the epoxide ring to form an ether linkage (C-O-C)^115^. These observations are consistent with the slight decrease in the intensity of the O-H band at 3347 cm^−1^ and the corresponding increase in the C-O band at 1030 cm^−1^ for CM-CF, as shown in Fig. 13a. For the CU-CF sample, an increase in the band intensity at 3347 cm^−1^ concerning FER can be observed. This may be attributed to potential chemical interactions between the hydroxyl groups of U-CF and the hydroxyl groups generated during curing, as well as the remaining epoxy groups of FER^116^. On the other hand, the functional groups introduced to the fiber surface by the polymeric coating are expected to improve the interaction with the PLA matrix^42^. In this regard, the carbonyl groups (C = O) observed at 1755 cm^−1^ in the FTIR spectrum of PLA could interact with the hydroxyl groups (O-H) of the coated fibers through hydrogen bonding^117,118^. This can improve the adhesion between the polymer matrix and the FER coating.

### Contact angle measurement

The contact angle (CA) can be an effective indicator of surface hydrophilicity, given that it is directly related to surface energy^119^. This parameter is useful for evaluating the compatibility and wettability of fibers within a matrix^119,120^. Contact angle measurements for U-CF, M-CF, CU-CF, and CM-CF using deionized water as the test liquid are shown in Fig. 13b. The mean CA of U-CF was 107.30 ± 6.08°, whereas that of M-CF was 108.11 ± 2.15. The results suggest that the alkali treatment did not alter the surface hydrophobicity of the CF. However, the treated fiber showed less variability than U-CF did, as indicated by its greater standard deviation (error bar). Compared with other fibers, CF has a greater CA due to its high content of nonpolar material (i.e., lignin and impurities) on its surface^121,122^. After alkaline treatment, the dissolution of noncellulosic materials such as lignin may improve the homogeneity of the CF contact surface area^19,123^. SEM and FTIR results revealed that M-CF exhibited a rougher yet more uniform surface and a diminished concentration of polar hydroxyl groups. This suggests that both the surface roughness and the homogeneity of the contact surface area in M-CF may contribute to the reduced variability in contact angle measurements. Consequently, the wettability of M-CF with the test liquid becomes more homogeneous. After coating, the hydrophobicity of both fibers decreased. The CA of CU-CF was 94.50 ± 1.78°, ~ 12% lower than that of U-CF. Similarly, the CA of CM-CF was 94.55 ± 3.01°, ~ 13% lower than that of M-CF. This decrease in hydrophobicity is likely caused by the presence of new functional groups on the fiber surface introduced by the FER coating. Functional groups such as hydroxyl (O-H), ether linkages (C-O-C), and residual epoxy groups can modify the surface energy of the CFs. According to the literature, these functional groups can slightly increase the polarity of the fiber^124^. Consequently, the interaction between the coated fibers and the polar groups in the test liquid is enhanced. The CA values for the coated samples were closest to the contact angle of PLA (80.0°)^119^. In terms of wettability, a small difference between the contact angle of the matrix and the fiber suggests an improvement in the interaction between both materials, as increased wettability enhances compatibility^119^. Consequently, PLA impregnation in the coated fibers is expected to be more efficient, improving fiber‒matrix adhesion.

## Single-fiber tensile test

**Fig. 14:**
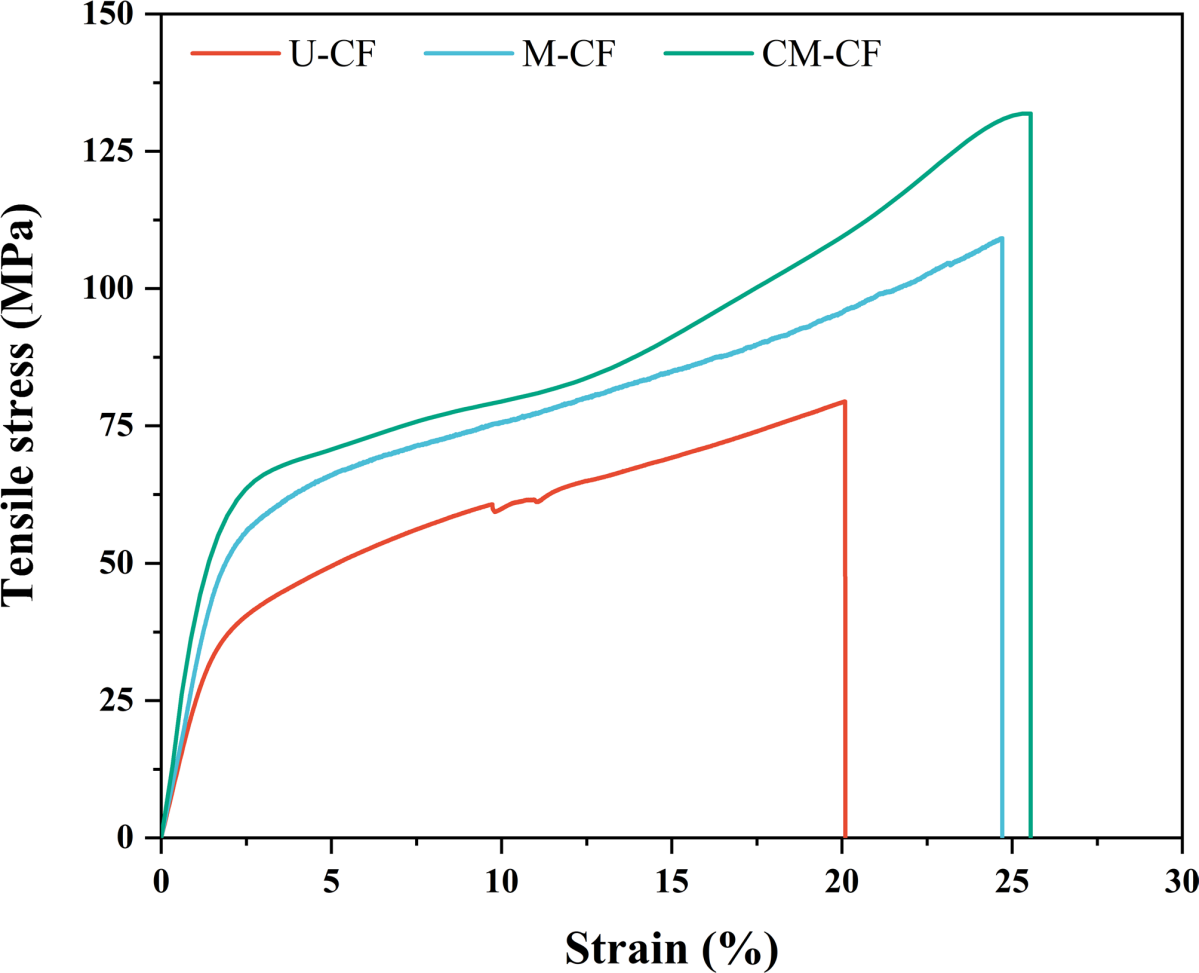
Representative tensile stress-strain curves of coir fibers: untreated (U-CF), mercerized (M-CF), and coated-mercerized (CM-CF).

**Fig. 15:**
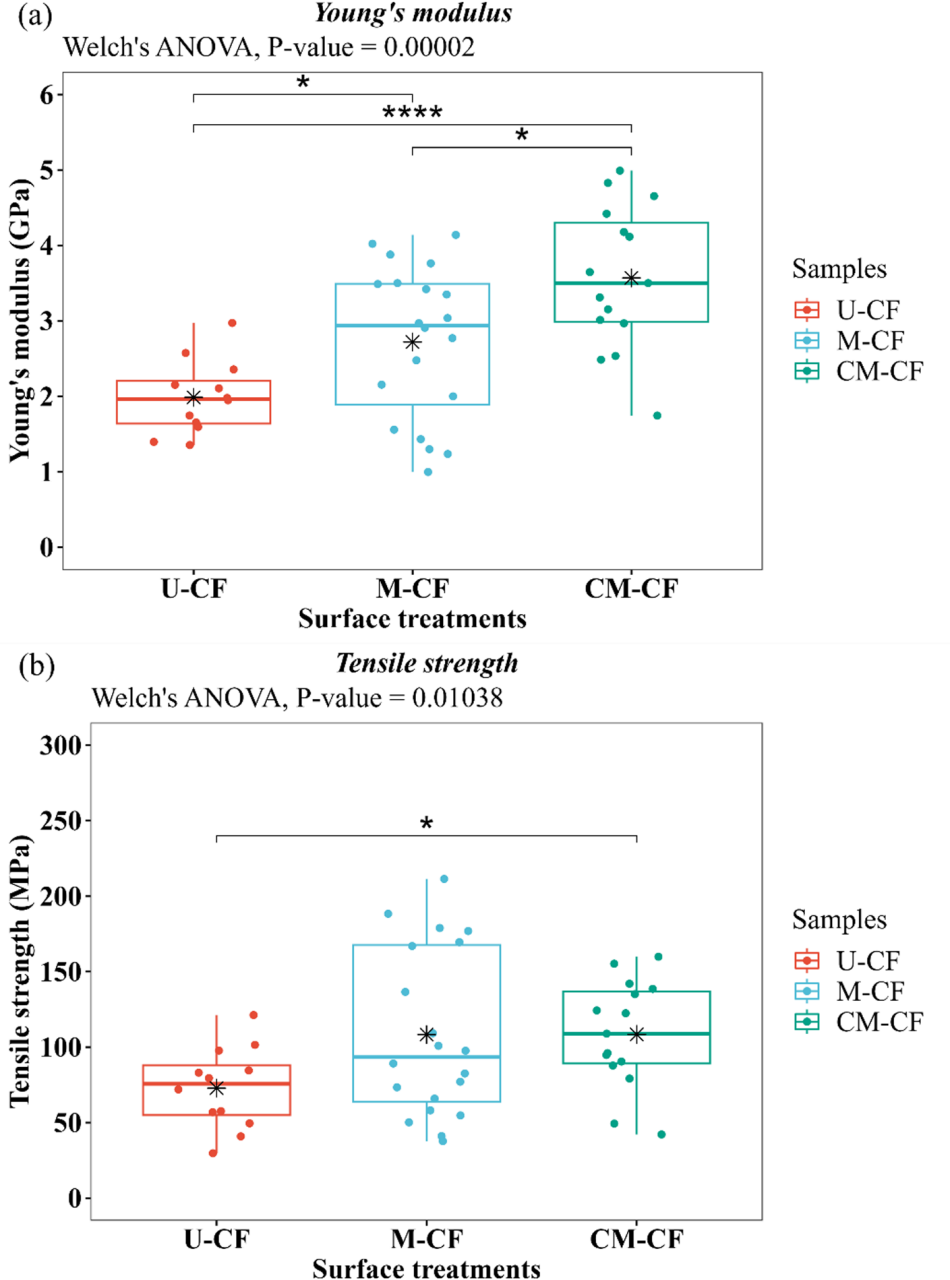
Mechanical properties of samples with different surface conditions. The black star-shaped symbol represents the mean of each Box plot. Significant difference at a 95% confidence level: (*) *P* value < 0.05 and (****) *P* value < 0.0001. Using R (version 4.2.2. https://www.r-project.org/).

Figure 14 shows the representative stress‒strain curves for each fiber under different surface conditions: U-CF, M-CF, and CM-CF. The curves of M-CF and CM-CF exhibit behavior similar to that of U-CF, as described in the subsection “Analysis of the tensile properties of dried coir fibers”. However, these samples showed a more pronounced slope at the beginning of the linear region of loading. Additionally, the tensile strength and elongation of the CFs increased after mercerization and coating. According to Ferreira et al.^125^ and Leites et al.^126^this is attributed to the removal of noncellulosic components during mercerization, which leads to the formation of interfibrillar regions that promote deformation in the fibers. Additionally, flexible polymeric coatings can provide fibers with a rigid yet flexible structure^105^. Tables S2–S4 in Annexure II of the supplemental material provide details on the descriptive and inferential statistical analyses. The normality of the data was assessed via the Shapiro–Wilk test and QQ plot (see Figure S3 and Table S3 in Annexure II of the supplementary material). Two mechanical properties had *P* values > 0.05, and their standardized residuals aligned with the dashed diagonal in the QQ plots, confirming that the normality assumption was satisfied. However, Levene’s test revealed *P* value < 0.05 for UTS, confirming that the assumption of equal variances was not met (Table S3 in Annexure II of the supplementary material). Therefore, Welch’s ANOVA was performed. Figure 15 shows the analysis of Young’s modulus and UTS results for samples with three different surface conditions. The results presented in Fig. 15a show the effects of surface conditions on the Young’s modulus of the CFs. Welch’s ANOVA revealed a significant difference between surface conditions (*P* value = 0.00002), and Games–Howell post hoc comparisons confirmed multiple significant differences among groups (see Table 8). The modulus was lowest for the untreated fibers (U-CF: 2.21 ± 0.92 GPa). Mercerized fibers (M-CF) exhibited an ~ 23% increase in stiffness (2.72 ± 1.01 GPa), whereas the CM-CF samples achieved an ~ 62% improvement over U-CF, with a modulus of 3.57 ± 0.95 GPa. In particular, CM-CF showed highly significant differences compared with U-CF (*P* value = 0.00003), indicating a positive effect of the combination of mercerization and coating on CF stiffness. The removal of noncellulosic materials, such as lignin, hemicellulose, and impurities, may increase the relative cellulose content in M-CFs. Cellulose is well known for imparting strength and stiffness to LFs^126^. This would explain the difference between U-CF and M-CF. Furthermore, mercerization induces structural modifications. These include higher crystallinity and a reduced microfibrillar angle, contributing to increased stiffness^86,126^. However, this treatment also introduces surface defects by removing the outer fiber layer. Upon coating and mercerization, the epoxy resin impregnates the fiber surface, filling pits and defects from chemical treatment. SEM analysis (Fig. 12) revealed that the CM-CF had a smoother and more even surface, as mentioned in the “Scanning electron microscopy (SEM)” section. This is likely because the polymeric coating acts as a stabilizing agent for the fiber structure. It prevents deformation and subsequent collapse of the cellulose microfibrils and increases their rigidity^39^. Figure 15b shows the UTS results for the fibers under different surface conditions. Welch’s ANOVA revealed significant differences among the groups, with a *P* value of 0.01038 (see Table S4 in Annexure II of the supplementary material). These results indicate that the surface conditions of the CFs influenced the tensile strength of the fibers. However, the Games–Howell test revealed that only the comparison between the U-CF and CM-CF samples was statistically significant (*P* value < 0.05) (see Table 8). The U-CF samples presented the lowest UTS values (75.85 ± 26.94 MPa), whereas the CM-CF samples presented an increase of ~ 43% in the mean value (108.43 ± 35.72 MPa). This enhancement may be due to the polymeric coating assisting in stress transfer in the hierarchical structure of the fibers, preventing premature failure and increasing the strength of the fibers^38^. Furthermore, as mentioned in the “Preparation of CF Surfaces Under Different Conditions by Mercerization and Polymeric Coating” subsection, the mean coating thickness was 25.59 ± 12.44 μm. This value is smaller than the mean U-CF diameter of 316.00 ± 86.42 μm, representing only ~ 8% of the total diameter. Therefore, it can be inferred that the polymeric coating is primarily a layer around the fiber rather than a matrix. Notably, no statistically significant difference was observed between M-CF and CM-CF. Nevertheless, M-CF presented a greater UTS scatter, as indicated by the longer box-and-whisker plot. This may be due to the greater microstructural heterogeneity caused by the chemical treatment, which induced the elimination of surficial impurities and possibly cellulose chain cross-linking^19^. In contrast, the CM-CF samples exhibited lower data scattering. The results suggest that the coating compensates for heterogeneities introduced by the chemical treatment, allowing the tensile strength of the fiber to be preserved^127^. Similar behavior has also been reported by^128^. Hence, the tensile test results suggest that the mercerizing and coating steps improve the mechanical response of the CFs.

**Table 8:** Results of mean comparison: Games–Howell pairwise comparison of tensile properties among the different surface conditions.

Games-Howel Pairwise means Comparison	Young’s modulus			Tensile strength		
α = 0.05	Means difference	*P* value	Significance	Means difference	*P* value	Significance
CM-CF vs. M-CF	−0.849	0.042	*	−0.105	1.0	ns
CM-CF vs. U-CF	−1.58	0.00003	****	−35.6	0.018	*
M-CF vs. U-CF	−0.734	0.026	*	−35.5	0.055	ns

Significant difference at a 95% confidence level: (*) *P* value < 0.05 and (****) *P* value < 0.0001. ns: non-significant difference.

### Single-fiber pull-out test

**Fig. 16:**
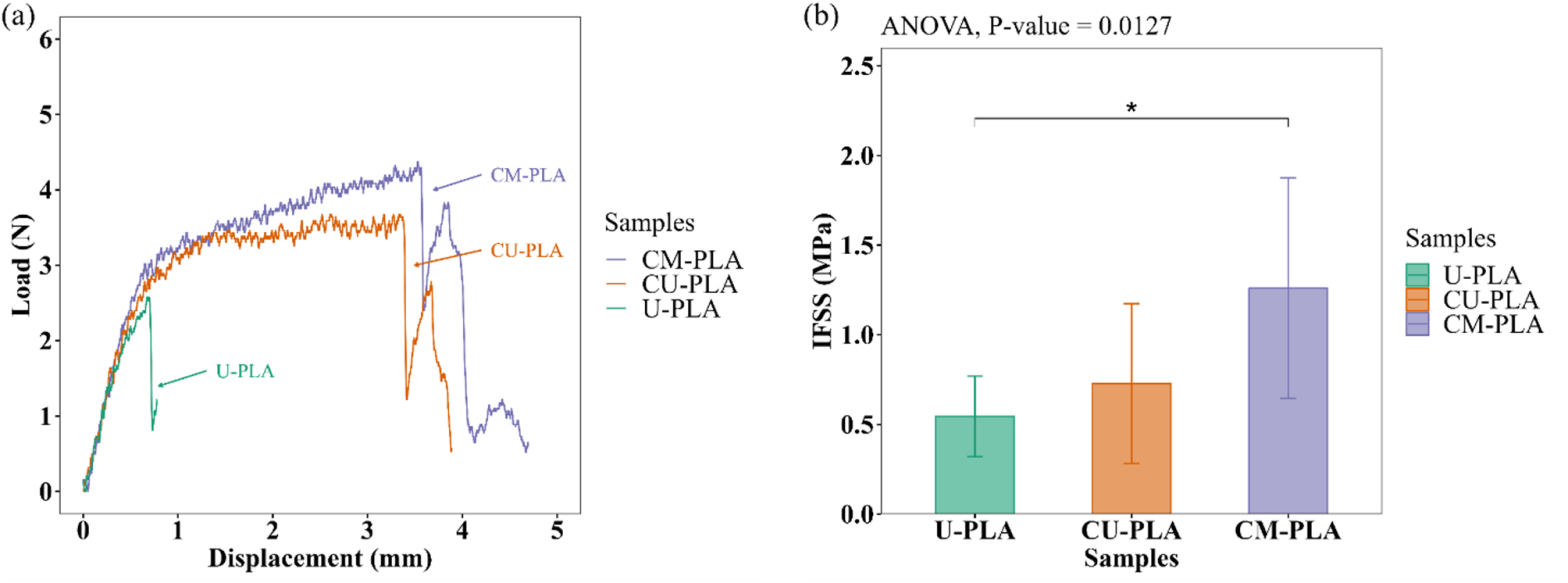
**a** Representative load-displacement curves of the groups: U-PLA, CU-PLA, and CM-PLA, and **b** Bar graphs showing the One-way ANOVA statistical analysis for the groups. Significant difference at a 95% confidence level: (*) *P* value < 0.05. Using R (version 4.2.2. https://www.r-project.org/).

Table 9 summarizes the pull-out test results for CFs with different surface conditions on the FER and PLA matrices. During the tests, some samples fractured outside the embedded length, reducing the number of pulled-out samples. Consequently, for each sample, the number of successful tests (n), the mean maximum load, the mean IFSS, and their respective standard deviations were reported. For the FER matrix samples, the IFSS was 0.709 MPa for U-CF (*n* = 1) and 0.946 ± 0.262 MPa for M-CF (*n* = 5). This difference can indicate that surface treatment may improve fiber‒matrix adhesion. However, a statistical analysis was not performed because of the insufficient number of pulled-out samples. Future studies should evaluate the effects of mercerization treatment on the IFSS of these samples, ensuring a sufficient sample size for statistical reproducibility. Compared with the U-PLA samples, the M-PLA samples presented a slight increase in the mean IFSS (0.916 ± 0.164 MPa). This suggests that mercerization facilitates mechanical interlocking with the PLA matrix^8^. However, this group was excluded from the statistical analysis because of its small sample size (*n* = 3). Therefore, for the PLA matrix samples, one-way ANOVA was performed to compare U-PLA, CU-PLA, and CM-PLA. In this regard, the effects of coating and pre-mercerization on the interfacial adhesion between CF and PLA were evaluated. Figure 16a displays representative load‒displacement curves during the fiber pull-out process for the U-PLA, CU-PLA, and CM-PLA samples. The curves clearly exhibit a trend in the behavior of the loading process across the displacement range. The fiber is loaded to a maximum value. At this point, complete fiber-matrix debonding occurs, leading to a sudden drop in load during pull-out. As shown in Table 9, the U-PLA samples attained lower load values than did the coated fibers, i.e., CM-PLA and CU-PLA. Moreover, the coated fibers sustained a load over a longer displacement, which likely indicates improved interfacial bonding performance between the CF and PLA. According to Ferreira et al.^125^polymeric coatings can act as bridges between fibers and the matrix, strengthening the interfacial bonding. Similarly, surface modification can alter the fiber pullout performance by changing the chemical and mechanical interactions between the fiber and matrix^39^. The latter is aligned with the potential interactions between FER and PLA, as evidenced by the FTIR spectra. In addition, it is supported by AC measurements that show improved wettability due to the increased polarity on the surface of the coated CFs.

**Table 9:** Pull-out test results of each sample and the number of pull-out samples performed by group.

Samples	No. of samples* (Counts)	Mean maximum load (*N*)	Standard deviation (MPa)	Mean IFSS (MPa)	Standard deviation (MPa)
U-FER	1	3.386	--	0.709	**--**
M-FER	5	5.633	1.650	0.946	0.262
U-PLA	8	1.764	1.095	0.544	0.225
M-PLA	3	2.702	1.434	0.916	0.164
CU-PLA	7	2.762	1.567	0.728	0.446
CM-PLA	9	3.765	1.714	1.260	0.615

Note: *No. of samples refers to the number of successful pull-out samples performed.

**Fig. 17:**
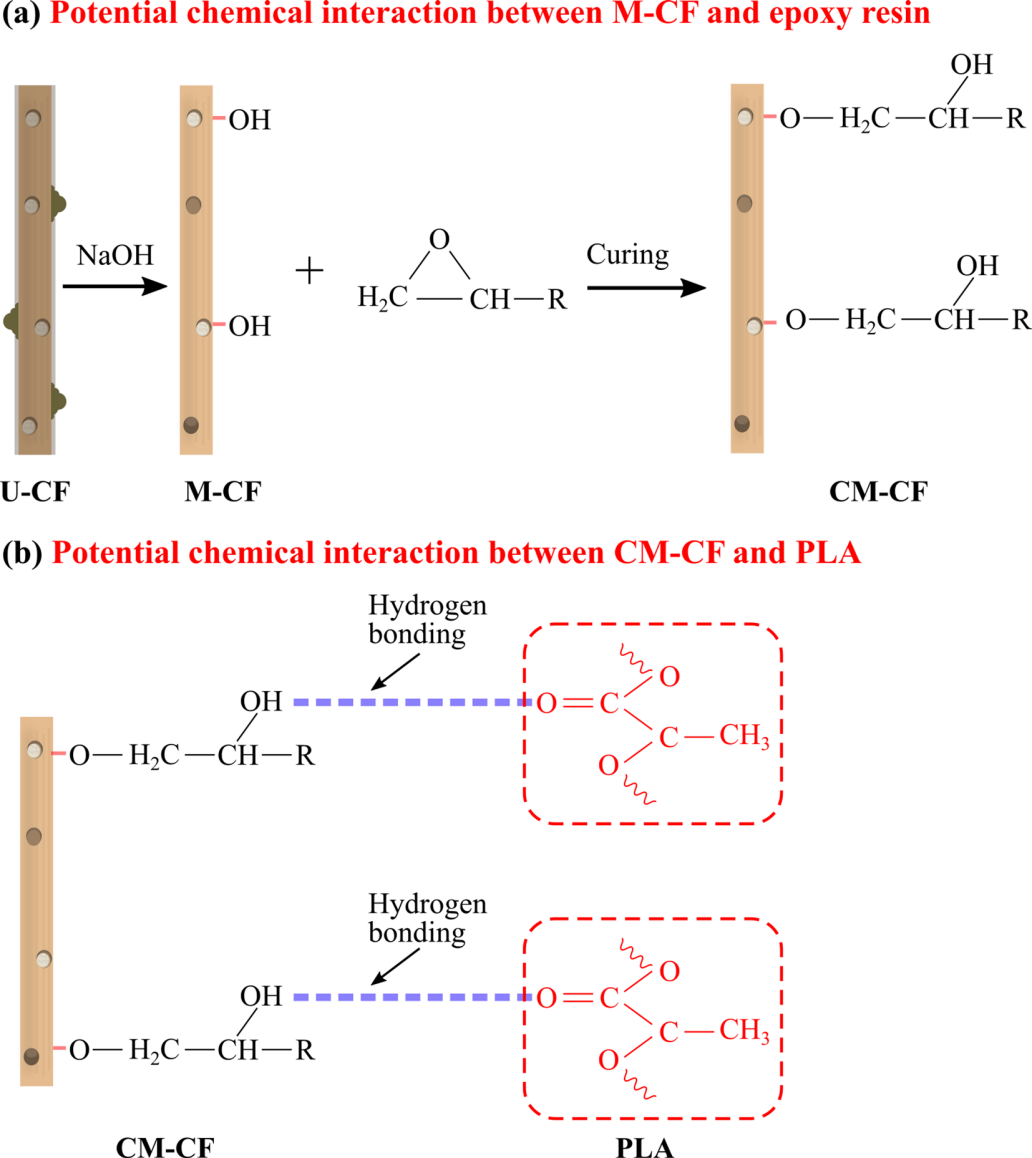
Possible chemical interaction between CF and different polymeric matrices.

**Fig. 18:**
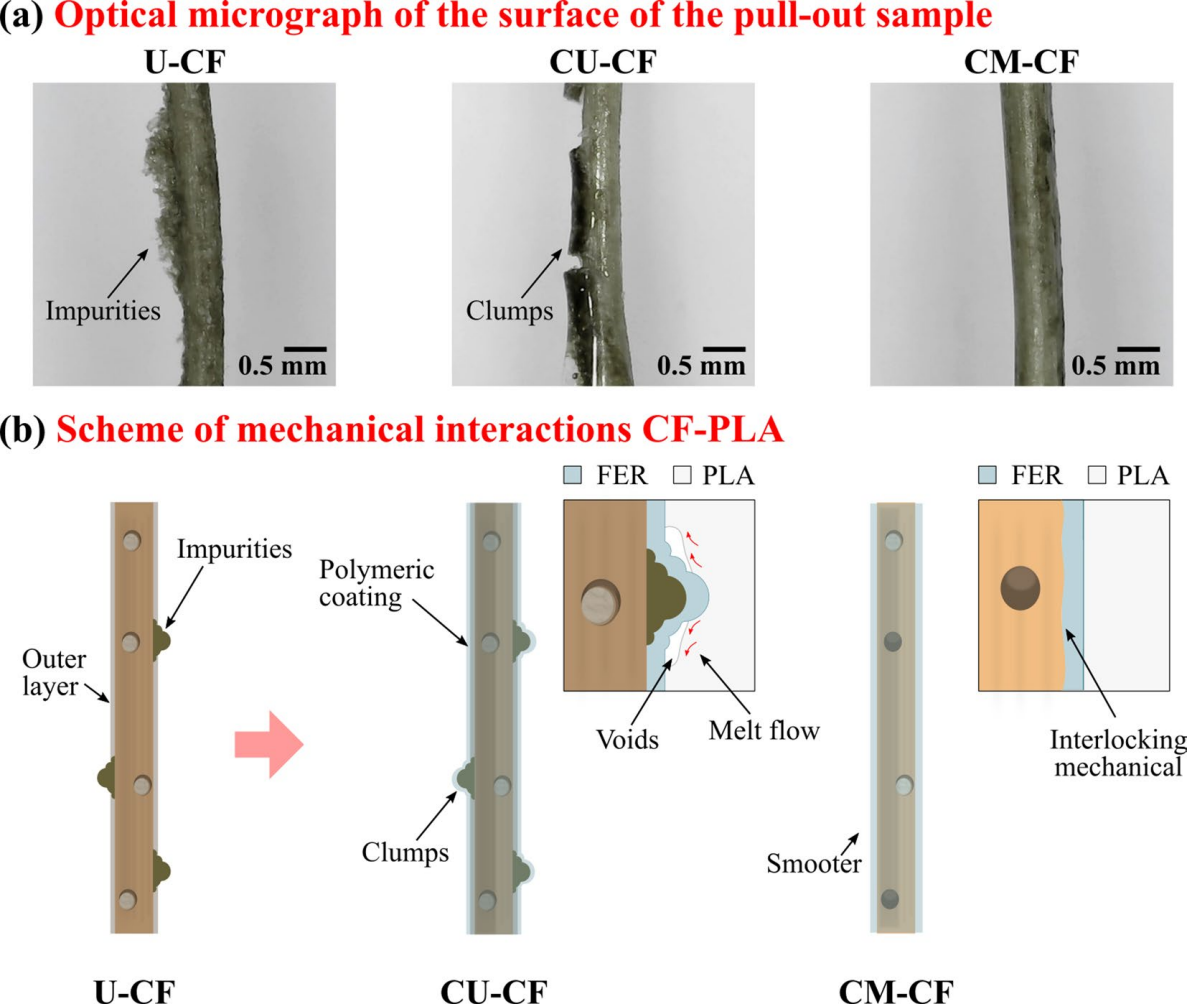
**a** Optical micrograph of surface morphology of the pull-out samples and **b** scheme of potential mechanical interactions between the coated fibers and the PLA matrix.

Figure 16b shows the statistical analysis of the IFSS for the three samples studied. A clear trend in the IFSS values from U-PLA to CM-PLA can be observed. Compared with the U-PLA samples, the CU-PLA samples presented an increase of 33%, whereas the CM-PLA samples presented a mean value that was twice as high. The Shapiro–Wilk test (*P* value > 0.05) and Levene’s test (*P* value > 0.05) confirmed that the assumptions of normality and homogeneity of variance were met (see Table S5 in Annexure II of the supplementary material). ANOVA yielded a *P* value = 0.0127, confirming statistically significant differences between the groups. However, post hoc Tukey tests (see Table 10) indicated that only the U-PLA and CM-PLA comparisons reached statistical significance (*P* value < 0.05). The tendency observed may be attributed to the possible chemical interactions between FER and the PLA matrix, resulting from secondary bonding formed between the C = O groups of PLA and the O-H groups of the cured epoxy resin^129^. This likely facilitates improved interfacial bonding in the PLA matrix and coated CF. However, the results suggest that mercerization before coating may enhance the interfacial adhesion between the CF and PLA matrix. During the pull-out test, load transfer is affected by the adhesion efficiency between the fiber and the coating layer. The bond between the FER coating and the matrix is expected to exceed the bond between the REF and the fiber^130^. It is believed that mercerization facilitates the mechanical interlocking of the fiber and the FER coating by partially removing noncellulosic materials from the fiber’s outer layer. Additionally, alkali treatment may generate reactive sites that expose cellulose, promoting chemical bonding through the opening of the epoxy ring during resin curing, as supported by FTIR analysis^115,131^. During NaOH treatment, non-cellulosic components are removed from the surface of U-CF, exposing the cellulose’s OH groups. When the epoxy resin is applied to M-CF, the OH groups can interact with the epoxide rings via ring-opening reactions during curing^113^. As a result, these interactions are believed to help form primary covalent bonds between fiber and FER. Figure 17a shows the proposed chemical interaction mechanism. After implementing the polymeric coating, the pull-out sample is prepared. The PLA matrix is then heated, melted, and extruded through the nozzle. Under these conditions, the mobility of the polymer chains may facilitate the formation of hydrogen bonds with the hydroxyl groups (-OH) of the coated fiber and the carbonyl groups (-C = O) of the PLA^117^as illustrated in Fig. 17b. This may result in improved interfacial bonding for the CM-PLA sample. On the other hand, the disposition of the polymeric coating on the surface can affect the contact area of the PLA matrix along the fiber length^105^. Figure 18a shows an optical micrograph of the surface morphology of the three analyzed samples. As mentioned in the subsection “Scanning electron microscopy (SEM)”, CM-CF has a smoother surface than CU-CF does, which facilitates better penetration and spreading of the PLA matrix, creating stronger interfacial bonding. This phenomenon is illustrated in Fig. 18b conceptually as a homogenous contact between the PLA and the fibers. In contrast, CU-CF exhibited irregularities (clumps) due to impurities on the untreated fibers (see Fig. 18a). The surface irregularities may hinder proper polymer impregnation, leading to weak adhesion zones or void formation during melt flow, as illustrated in Fig. 18b. During sample fabrication, PLA impregnation is limited by the low applied pressure and the viscosity of the polymer. According to Santos et al.^59^this manufacturing approach simulates the pull-out behavior of fibers in thermoplastic composites fabricated via 3D printing (see Fig. S2 in Annexure I of the Supplementary Material). Consequently, inadequate impregnation may result in the formation of voids at the fiber‒matrix interface^59^. Hence, the combination of mercerization treatment and FER coating represents a viable strategy to improve the mechanical properties of PLA/LF composites.

**Table 10:** Post hoc Tukey pairwise comparison of IFSS: U-PLA, CU-PLA, and CM-PLA samples.

Tukey Pairwise Means Comparison	IFSS		
α = 0.05	Means difference	*P* value	Significance
CU-PLA vs. CM-PLA	-0.533	0.0837	ns
U-PLA vs. CM-PLA	-0.716	0.0127	*
U-PLA vs. CU-PLA	-0.183	0.7321	ns

Significant difference at a 95% confidence level: (*) *P* value < 0.05. ns: non-significant difference.

### Feasibility of potential

This work shows the viability of Colombian coir fibers as a sustainable reinforcement for PLA matrix composites. These findings provide a feasible alternative to synthetic materials. The combination of physical and chemical treatments considerably enhanced the mechanical characteristics and interfacial adhesion of the CF and PLA matrix. Drying at 90 °C decreased the moisture content and enhanced the fiber stiffness, and mercerization eliminated surface impurities from the outer layer, increasing the roughness for better mechanical interlocking. Moreover, the FER coating chemically modified the surface of the fibers, slightly increasing their polarity. Furthermore, this polymeric coating promoted chemical interactions with the PLA matrix. In terms of the mechanical properties, the combined drying, mercerization, and polymeric coating increased the Young’s modulus by 62% and the tensile strength by 43%. Similarly, the pull-out results revealed that the mean IFSS value doubled compared with that of the untreated fiber (U-CF), confirming stronger adhesion between the modified CF (CM-CF) and PLA. As a result, the approaches employed are practical, scalable, and based on easily available methodologies, facilitating their implementation. Compared with treatments such as silane, acetylation, and plasma treatment, which typically require the use of expensive materials, complex instrumentation, and multistep procedures^39^FER coatings provide a simpler and lower-cost alternative for surface modification of LFs. Moreover, beyond technical advances, the low density of CF (0.65 g/cm³) and its origin as agro-industrial waste fit with circular economy concepts, supporting the sustainable use of the material.

Although this study did not examine the properties of a CF-reinforced PLA composite, the different treatments examined have great potential for use in manufacturing 3D-printed composites. PLA is the most widely used thermoplastic polymer for 3D-printing composites with the fused filament fabrication (FFF) technique^21^. The IFSS results demonstrate that the combined mercerization and FER coating treatments significantly improve fiber-matrix adhesion between CF and PLA. CM-PLA achieved a mean IFSS of 1.260 MPa, compared to just 0.544 MPa for U-PLA. These findings suggest that these treatments could facilitate more efficient stress transmission in PLA matrix composites. Furthermore, the epoxy coating forms a new layer on the surface fiber, conforming to fiber morphology. Additionally, the FER coating creates a new layer on the fiber’s surface that conforms to its morphology. The epoxy coating covers the fiber’s surface imperfections and irregularities. Furthermore, the FER provides new functional groups on the fiber surface, decreasing the AC from 107.30° for U-CF to 94.55° for CM-CF. This could facilitate impregnating the fibers with the molten matrix during 3D printing. This is particularly important for twisted continuous yarns because their morphology promotes void formation and hinders matrix infiltration during deposition^59^. Furthermore, this modification may reduce the risk of nozzle clogging and promote better fiber dispersion in the extruded matrix, which is essential for uniform extrusion.

## Conclusions

This study evaluated the effects of drying temperature, mercerizing, and epoxy coating on the physicochemical and mechanical properties of Colombian coconut fibers and their interfacial adhesion with PLA. Drying analysis revealed that the CF had a higher moisture removal rate at 90 °C. Morphological changes in the CFs were observed via SEM. Microcracks and partial surface degradation were found in the CFs dried at 90 °C, which might be attributed to moisture loss and volatile evaporation during the drying process. The FTIR spectra revealed changes in the absorption bands after drying at 90 °C, indicating the removal of the fiber degradation products. According to the mechanical property analysis, the drying temperature affected the stiffness of the CFs. This was associated with moisture loss, which can cause a shift from ductile to brittle behavior, hardening the fibers. Mercerization and polymeric coating considerably enhanced the mechanical properties of the CFs. Mercerization partially removed noncellulosic components, and the coating filled the resulting surface defects. This resulted in a 62% increase in the stiffness and a 43% increase in the strength, as well as greater uniformity in the properties. CA analysis revealed that mercerization did not affect the hydrophobicity of the CFs; however, it reduced the measurement variability owing to the surface roughness and homogeneity of the M-CF contact surface area. The FER coating introduced polar functional groups that reduced hydrophobicity and potentially improved wettability with PLA. Pull-out tests revealed that the polymeric coating significantly enhanced the interfacial adhesion between CF and the PLA matrix, doubling the IFSS for CM-PLA compared with U-PLA. Mercerization before coating promoted mechanical and chemical interactions by removing impurities and exposing reactive groups. On the other hand, the FER coating acted as a bonding bridge with the matrix. However, surface irregularities hinder the adhesion of the CU-PLA.

## Supplementary Information

Below is the link to the electronic supplementary material.

Supplementary Material 1

## Data Availability

The data related to this work can be obtained from the corresponding author by email request.
