# Supplementary material for: Effects of drying temperature, mercerizing, and coating on the properties of Colombian Coir fibers and their interfacial adhesion with polylactic acid
Source: Sci Rep. 2025 Sep 2;15:32346. doi: 10.1038/s41598-025-18240-2 (PMC12405578; doi:10.1038/s41598-025-18240-2)
Supplement: Supplementary file 1 — Supplementary Material 1 [file 41598_2025_18240_MOESM1_ESM.docx]

**Supplementary material**

**for**

**Effect of Drying Temperature, Mercerizing, and Coating on the Properties of Colombian Coir Fibers and their Interfacial Adhesion with Polylactic Acid**

Yarley Buelvas Arrieta^1^, Linda Díaz Reyes^1^, César Ávila Díaz^1^, Juan Altamiranda Suárez^1^, Oswaldo Rivero-Romero^1,2*^, Jimy Unfried-Silgado^1^

^1^University of Cordoba, Mechanical Engineering Department, Research Group ICT, Monteria, Cordoba, Colombia.

^2^University of Antioquia, Mechanical Engineering Department, Research Group CCComposite, Medellin, Antioquia, Colombia.

* Corresponding author e-mail address: [oriveroromero@correo.unicordoba.edu.co](mailto:oriveroromero@correo.unicordoba.edu.co), https://orcid.org/0000-0002-3451-7409

Address: Cra 6 No. 77- 305 Montería - Córdoba, Colombia;

ZIP: 230002; PBX: +57(4)7860920.

**Annexure I**

1. **Single-fiber pull-out specimens’ preparation**

**1.1. Epoxy resin matrix**

Epoxy resin (ER) specimens were fabricated using a silicone mold with a 10 × 10 mm cavity and a depth of 4.0 mm. The coir fibers (CFs) were cut into 50 mm segments and fixed to the base of the mold. The CFs were straightened and secured to an acrylic frame using fixed tape to ensure proper alignment. Next, a 1:1 mixture of resin and hardener was poured into the mold. Once the mold was filled, the samples were left to cure for 24 hours at room temperature. Figure S1(a) illustrates the molding process for the specimens intended for the pull-out test. After demolding, the bottom surface of the samples was sanded with 600-grit sandpaper to remove any excess fiber protruding from the mold. This resulted in prismatic specimens measuring 10 × 10 × 4 mm (see Fig. S1(b)), with a controlled embedded length of 4 mm. For the tensile test, a free fiber end of 30 mm was maintained above the specimen's central point. Figure S1(c) shows the final specimen.


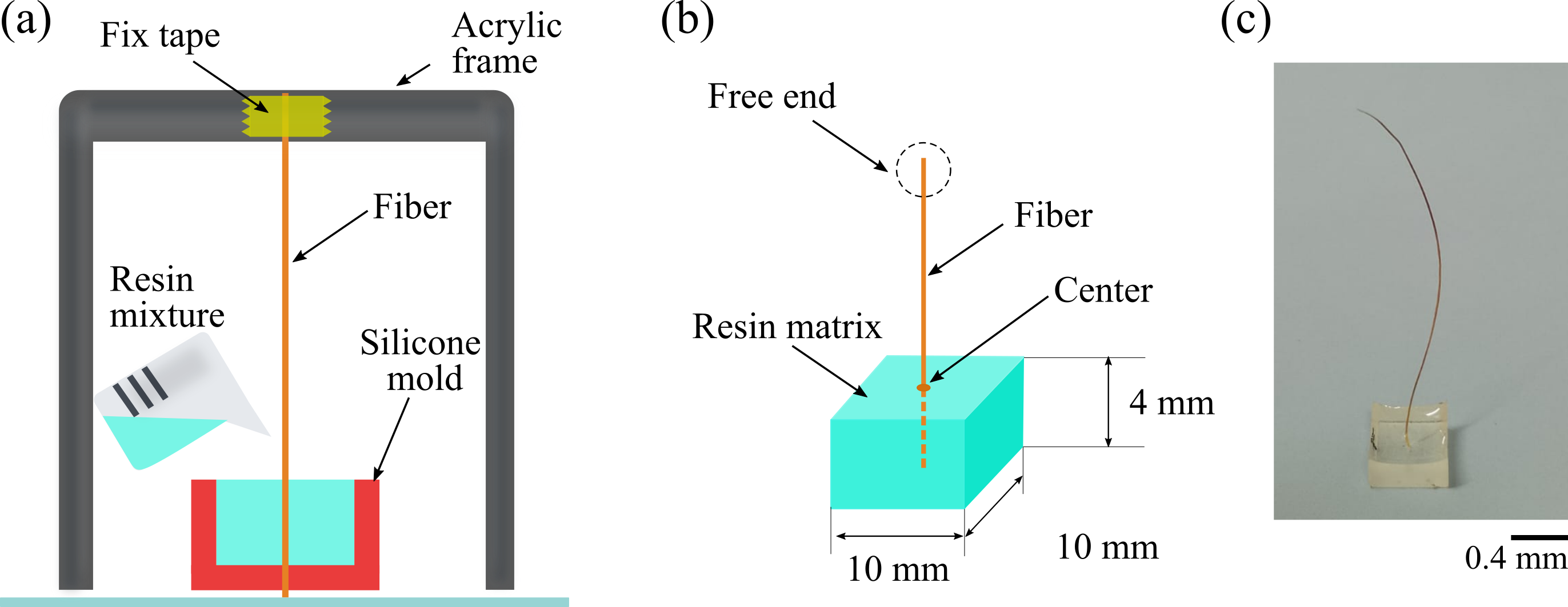


**Fig. S1:** Preparation of samples with ER matrix: (a) molding process of the pull-out specimens, (b) schematic representation of the pull-out configuration, and (c) final specimen.

**1.2. Polylactic acid matrix**

The polylactic acid (PLA) matrix samples were fabricated using a fused filament fabrication (FFF) 3D printer via layer-by-layer deposition. Several authors previously reported this methodology ^1,2^. The preparation method consists of depositing 2 layers of PLA (Fig. S2(a)), pausing the printing process to position the fiber along it (Fig. S2(b)), and finally, 2 additional layers were printed (Fig. S2(c)). This ensures that the molten matrix is deposited to encapsulate the fiber within the interlayer junction fully^1^ (see Fig. S2(d)). A total of 4 layers were 3D printed. Table S1 summarizes the main printing parameters used. Sample geometry, including the hole, was defined as a CAD model (Fig. S2(e)). The embedded length was controlled by cutting the fiber at the top and bottom edges of the hole. 4 mm was maintained from the fiber entry point to the top edge of the hole, corresponding to the embedded length. For the pull-out test, the fibers were gripped 30 mm above the entry point. The implemented method allows simulating the pull-out behavior of natural fibers in thermoplastic composites produced via FFF ^1^.

**
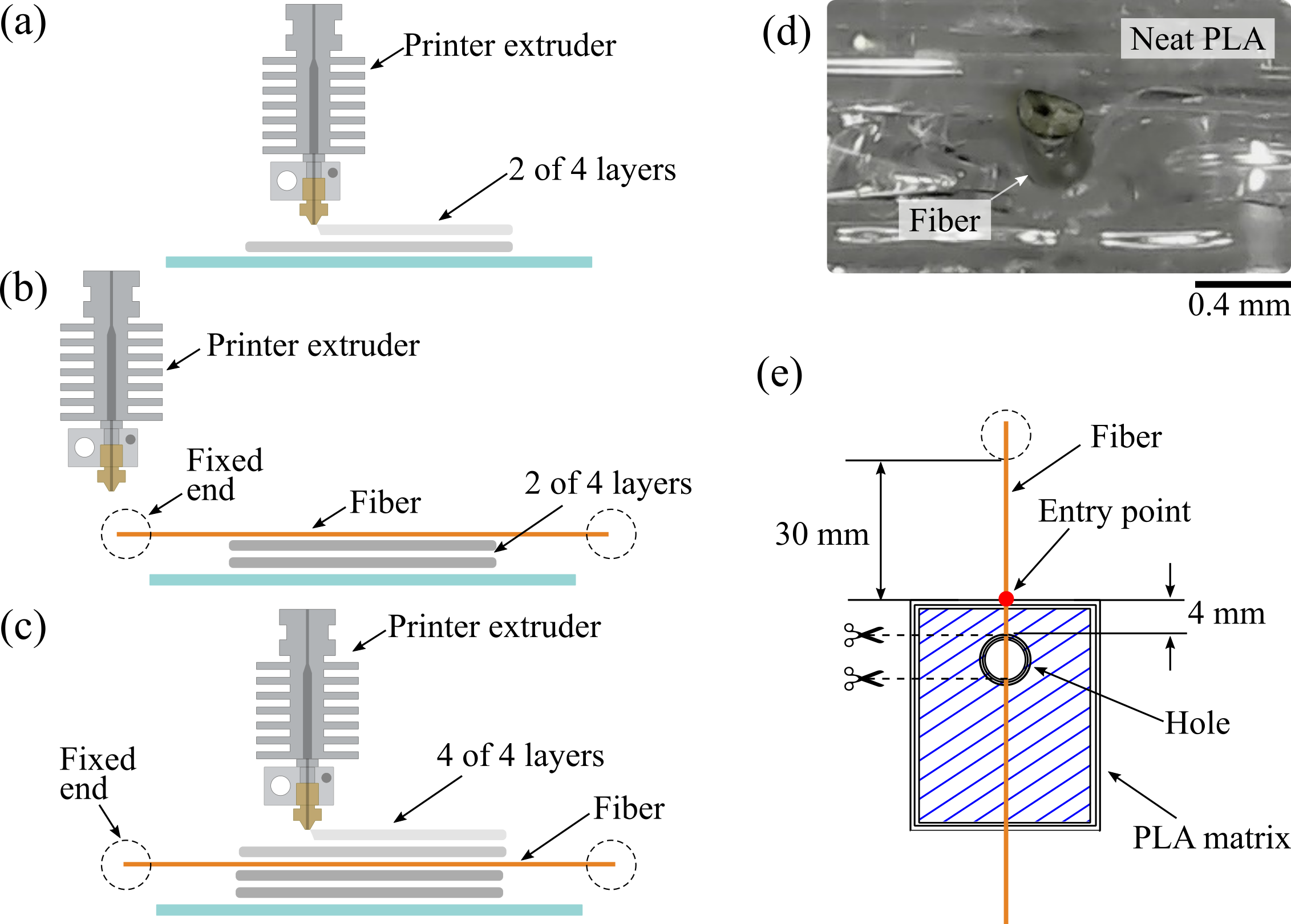
**

**Fig. S2:** Preparation of pull-out test specimen with PLA matrix: (a) printing of the initial layers, (b) placement of the coir fiber, (c) completion of the remaining layers, (d) close-up of PLA deposition, and (e) detail of the final specimen.

**Table S1:** Printing parameters used for manufacturing the pull-out samples.

| **Printing parameter** | **Values** |
| --- | --- |
| Nozzle diameter | 0.6 mm |
| Printing temperature | 210 °C |
| Bed temperature | 60 °C |
| Printing speed | 300 mm/min |
| Layer width | 0.6 mm |
| Layer thickness | 0.4 mm |
| Raster angle | 45° |

**Annexure II**

1. **Statistical data on the mechanical properties and pull-out**

**2.1. Statistical Analysis on the tensile properties of fibers under different surface conditions**

Table S2 shows the descriptive statistics for mechanical properties, i.e., Young's modulus and ultimate tensile strength (UTS), for the three sample groups: untreated fiber (U-CF), mercerized coir fiber (M-CF), and coated and mercerized coir fiber (CM-CF). The results show that the CM-CF group exhibits the highest mean Young's modulus (3.57 MPa) and UTS (108.43 MPa), followed by the M-CF group (2.72 MPa and 108.32 MPa, respectively). The U-CF group displays the lowest values (2.21 MPa and 75.85 MPa). Notably, the standard deviations for UTS are higher in M-CF and CM-CF than in U-CF, suggesting greater variability in these groups.

**Table S2:** Statistical parameters for Young's modulus and tensile strength (UTS) of U-CF, M-CF, and CM-CF.

| **Samples** | **Mechanical properties** | **Sample size**  **(Counts)** | **Mean**  **(MPa)** | **Standard deviation**  **(MPa)** |
| --- | --- | --- | --- | --- |
| U-CF | Young’s modulus | 13 | 2.21 | 0.92 |
|  | UTS |  | 75.85 | 26.94 |
| M-CF | Young’s modulus | 20 | 2.72 | 1.01 |
|  | UTS |  | 108.32 | 55.32 |
| CM-CF | Young’s modulus | 15 | 3.57 | 0.95 |
|  | UTS |  | 108.43 | 35.72 |

The normality assumption was examined through residuals instead of raw data. According to authors such as Roncho et al.^3^, evaluating the normality of residuals controls the type I error rate. The Shapiro-Wilk normality test is shown in Table S3. The Shapiro-Wilk test (*P* value > 0.05) confirms that the residuals for both Young's modulus and UTS follow a normal distribution. This can be corroborated by the QQ-plots shown in Figure S3. As can be seen, the standardized residuals are adjusted with the dashed diagonal line, indicating that the data follows a normal distribution. On the other hand, Levene's test results for examining homogeneity assumption can be seen in Table S3. Levene's test indicates a violation of variance homogeneity for both properties (*P* value = 0.040 and *P* value = 0.037, respectively). This necessitates the use of Welch's ANOVA to account for heteroscedasticity.

**Table S3:** Statistical tests of normality and variance homogeneity for One-Way ANOVA residuals for tensile results of U-CF, M-CF, and CM-CF (*P* value > 0.05, there is insufficient evidence to reject normality and homogeneity).

| **Statistical tests** | **Young’s modulus** | | **UTS** | |
| --- | --- | --- | --- | --- |
| **α = 0.05** | **Statistics** | ***P* value** | **Statistics** | ***P* value** |
| Shapiro-Wilk’s test | 0.973 | 0.339 | 0.975 | 0.419 |
| Levene’s test | 3.476 | 0.040* | 3.565 | 0.037* |

The symbol (*) indicates a significant difference at a significance level of 95%.


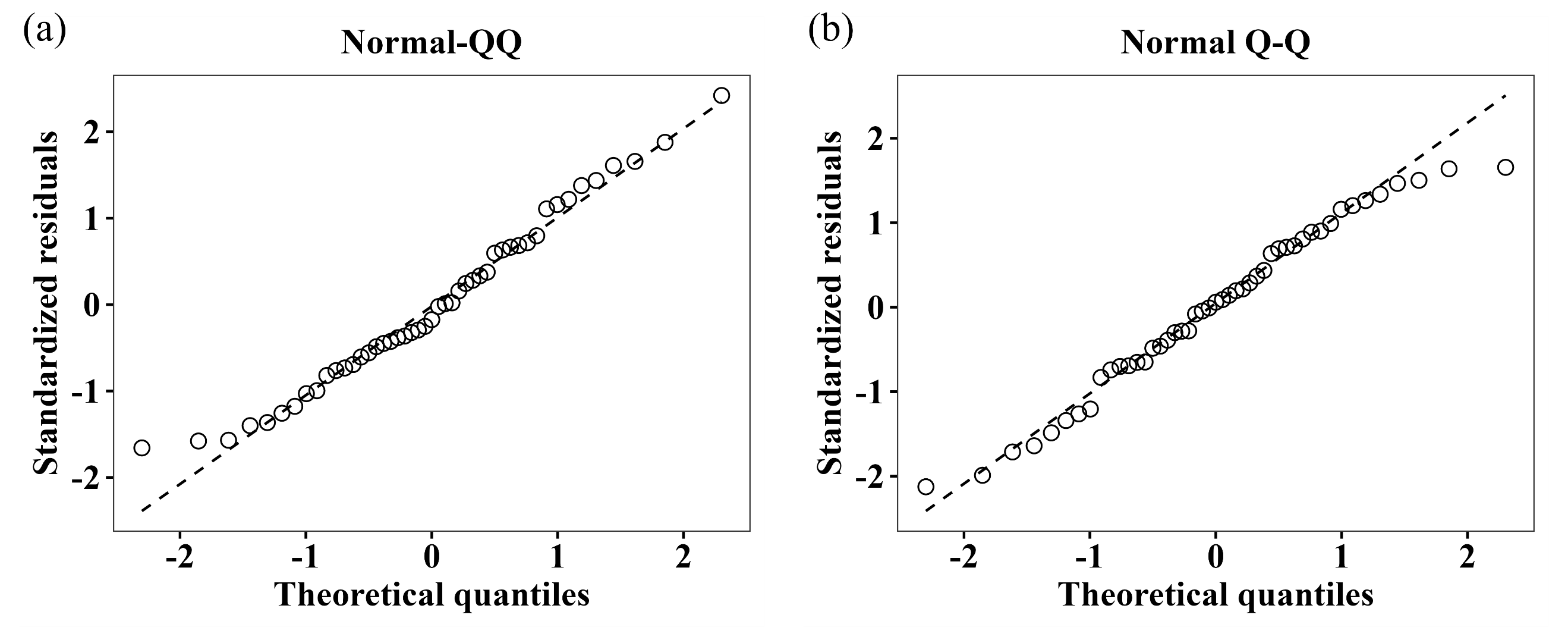


**Fig. S3:** Normal Q-Q plot of residuals: (a) Young’s modulus and (b) Tensile strength.

Table S4 summarizes the results of Welch’s one-way ANOVA, revealing statistically significant differences in both Young's modulus (F = 16.244, *P* value < 0.001) and UTS (F = 5.361, *P* value = 0.010) among the groups. These findings suggest that the surface modifications significantly influence the mechanical properties of CFs.

**Table S4:** Statistical tests for U-CF, M-CF, and CM-CF tensile properties.

| **Mechanical properties** | **Statistical tests (α = 0.05)** | ***F* value** | ***P* value** | **Remarks** |
| --- | --- | --- | --- | --- |
| Modulus’s Young | Welch’s One-way ANOVA | 16.244 | 0.00002 | Significant |
| UTS | Welch’s One-way ANOVA | 5.361 | 0.01038 | Significant |

- 1. **Statistical analysis of data on pull-out tests**

Table S5 presents the statistical analysis of the interfacial shear strength (IFSS) for the following samples: untreated fiber embedded in PLA (U-PLA), coated untreated fiber embedded in PLA (CU-PLA), and coated mercerized fiber embedded in PLA (CM-PLA). The normality assumption was examined using residuals. The Shapiro-Wilk test confirmed that the residuals follow a normal distribution (*P* value = 0.6628), and Levene’s test indicated homogeneity of variances (*P* value = 0.2419). These results validate the assumptions for one-way ANOVA. The ANOVA results revealed a statistically significant difference in IFSS among the groups (F = 5.411, *P* value = 0.0127). This suggests that surface modifications affected interfacial adhesion with the PLA matrix.

**Table S5:** Statistical tests for IFSS of U-PLA, CU-PLA, and CM-PLA.

| **Statistical tests (α = 0.05)** | **Statistics** | ***P* value** |
| --- | --- | --- |
| Shapiro-Wilk’s test | 0.9698 | 0.6628 |
| Levene’s test | 1.5195 | 0.2419 |
| One-way ANOVA | 5.411 | 0.0127* |

The symbol (*) indicates a significant difference at a significance level of 95%.

1. **References**

1. dos Santos, N. V. et al. Analysis of voids, interfacial and thermal properties of additively manufactured continuous natural fiber-reinforced biocomposites. Prog Addit Manuf (2024). <https://doi.org/10.1007/s40964-024-00913-5>.

2. Dange, D. & Gnanamoorthy, R. Effect of alkaline treatment of coir fibre on the interfacial adhesion in coir fibre-reinforced polylactic acid bio-composite. Mater. Today Proc., 2214-7853 (2023).

3. Rochon, J., Gondan, M. & Kieser, M. To test or not to test: Preliminary assessment of normality when comparing two independent samples. *BMC Med Res Methodol* **12**, 81 (2012). <https://doi.org/10.1186/1471-2288-12-81>.
